# Beyond α-Glucosidase and α-Amylase Inhibition: Integrated In Vitro and Multi-Scale In Silico Insights into the Antidiabetic and Antioxidant Mechanisms of *Oxalis corniculata* L. Aerial Parts

**DOI:** 10.3390/molecules31040630

**Published:** 2026-02-12

**Authors:** Thi-Van-Anh Nguyen, Duong Quang Quy, Nguyen Thanh Tung, Nguyen Thu Huyen, Pham Le Minh, Nguyen T. Minh Huyen, Tue-Tam Ho, Nguyen Thi Thu Ha, Gerardo M. Casanola-Martin, Bakhtiyor Rasulev, Hai Pham-The

**Affiliations:** 1 Department of Life Sciences, University of Science and Technology of Hanoi (USTH), Vietnam Academy of Science and Technology, 18-Hoang Quoc Viet, Nghia Do, Hanoi 10000, Vietnam; nguyen-thi-van.anh@usth.edu.vn (T.-V.-A.N.); quang.duong@ndsu.edu (D.Q.Q.); huyennt.m23bio@usth.edu.vn (N.T.H.); pham-le.minh@usth.edu.vn (P.L.M.); nguyen-thi-minh.huyen@usth.edu.vn (N.T.M.H.); 2 Department of Coatings and Polymeric Materials, North Dakota State University, Fargo, ND 58102, USA; 3 Department of Pharmacognosy, Hanoi University of Pharmacy, 13-15 Le Thanh Tong, Cua Nam, Hanoi 10000, Vietnam; thanhtungng.pharmacist@gmail.com (N.T.T.); tama4k46@gmail.com (T.-T.H.); 4 Institute of Chemistry (ICH), Vietnam Academy of Science and Technology, 18-Hoang Quoc Viet, Nghia Do, Hanoi 10000, Vietnam; thuha.vast@gmail.com

**Keywords:** *O. Corniculata* aerial extract, antidiabetic and antioxidant activity, in vitro tests, polypharmacological network, molecular simulation, quantum chemistry

## Abstract

Diabetes mellitus is a major global health concern associated with severe metabolic and cardiovascular complications. This study evaluated the antidiabetic and antioxidant activities of *Oxalis corniculata* L. aerial parts, with a focus on α-glucosidase and α-amylase inhibition, using a combination of in vitro assays and in silico analyses. Among the tested fractions, the ethyl acetate fraction exhibited the strongest inhibitory activity against both enzymes, with IC_50_ values of 0.097 and 0.015 mg/mL for α-glucosidase and α-amylase, respectively, surpassing those of the reference drug, acarbose. This fraction also demonstrated potent antioxidant activity, with IC_50_ values of 0.025 and 0.020 mg/mL in DPPH and ABTS assays, respectively. To elucidate the underlying mechanisms beyond digestive enzyme inhibition, bioactive constituents were screened and evaluated using network pharmacology, molecular docking, molecular dynamics simulations, and density functional theory (DFT) calculations. Molecular docking and dynamic simulations confirmed stable and energetically favorable interactions with α-glucosidase and α-amylase. Network pharmacology analysis revealed that the antidiabetic effects of *O. corniculata* involve modulation of insulin resistance-related pathways, particularly PI3K/Akt signaling, GLUT4 translocation, and inflammation-associated targets, alongside regulation of oxidative stress through redox-related enzymes. Complementary DFT analysis provided molecular-level insights into the antioxidant mechanisms, highlighting favorable electronic properties that support efficient radical scavenging. Overall, this integrated experimental–computational study provided valuable evidence of *O. corniculata* aerial parts as a promising multi-target phytotherapeutic candidate for diabetes management, extending its therapeutic relevance beyond α-glucosidase and α-amylase inhibition.

## 1. Introduction

Diabetes mellitus represents one of the most critical public health challenges of the 21st century, currently affecting more than 537 million adults worldwide, with prevalence expected to rise substantially in the coming decades [1]. Persistent hyperglycemia, the hallmark of diabetes, not only disrupts metabolic homeostasis but also contributes to the development of severe long-term complications, particularly cardiovascular diseases. Chronic exposure to elevated glucose levels induces endothelial dysfunction, low-grade inflammation, and excessive generation of reactive oxygen species (ROS), thereby accelerating oxidative stress-mediated vascular damage and increasing the risk of atherosclerosis, myocardial infarction, and stroke [2]. These interrelated pathological processes underscore the need for therapeutic strategies capable of simultaneously regulating postprandial hyperglycemia and mitigating oxidative stress.

Inhibition of α-glucosidase and α-amylase remains a well-established strategy for controlling postprandial hyperglycemia, while enhancement of antioxidant defenses is critical for reducing oxidative stress-driven diabetic complications, including cardiovascular disorders [3,4]. However, diabetes is increasingly recognized as a multi-factorial disease involving complex molecular networks rather than isolated targets [5]. Therefore, approaches capable of capturing multi-target interactions and pathway-level effects are essential for a comprehensive understanding of herbal antidiabetic agents.

Natural products derived from traditional medicinal plants continue to play a crucial role in the search for multi-target agents for metabolic disorders. *Oxalis corniculata* L., commonly known as creeping wood sorrel, has long been used in traditional medicine across Asia for the management of various ailments, including metabolic and cardiovascular disorders [6,7]. In Vietnam, particularly in the North Central region, the aerial parts of *O. corniculata* are widely consumed in folk medicine for the management of diabetes-related symptoms and cardiovascular health [8]. Phytochemical studies have reported that this species contains diverse bioactive constituents, such as flavonoids, phenolic acids, and polysaccharides, which are commonly associated with antioxidant and metabolic regulatory activities [9]. Despite the extensive traditional use of *O. corniculata*, systematic investigations linking its enzyme inhibition and antioxidant activity with molecular mechanisms are still lacking [10]. In particular, little attention has been paid to elucidating how individual phytochemicals contribute to multi-target antidiabetic and antioxidant effects at molecular and systemic levels.

In this study, dried aerial parts of *O. corniculata* collected from the North Central region of Vietnam were first extracted with ethanol, followed by successive solvent partitioning to obtain *n*-hexane, dichloromethane, ethyl acetate, and aqueous fractions, thereby enabling a comparative evaluation of bioactivities across fractions with different polarity profiles. We then investigated the antidiabetic and antioxidant properties of the *O. corniculata* L. extract/fraction through an integrated in vitro and multi-scale in silico framework. Initial enzyme inhibition assays targeting α-glucosidase and α-amylase, together with DPPH (2,2-diphenyl-1-picrylhydrazyl) and ABTS (2,2′-azinobis-(3-ethylbenzothiazoline-6-sulfonic acid)) radical scavenging assays, were employed. Subsequently, molecular docking and molecular dynamics simulations were conducted to elucidate ligand–enzyme interactions at the atomic level, while DFT calculations were applied to explore electronic features relevant to antioxidant mechanisms. Beyond classical enzyme inhibition, network pharmacology was utilized to identify additional diabetes- and oxidative stress–related targets, followed by molecular docking validation to assess multi-target interactions.

By integrating traditional knowledge, regional phytochemical relevance, and advanced computational analyses, this work seeks to move beyond isolated bioassays toward a systems-level understanding of the antidiabetic and antioxidant mechanisms of *O. corniculata* L. aerial parts. It should be emphasized that the present study is designed as a mechanism-oriented and hypothesis-generating investigation, in which biological activities are evaluated at the fraction level and representative phytochemicals reported in the literature are employed as a rational chemical space for multi-scale in silico analysis. Comprehensive phytochemical profiling and the isolation and in vitro validation of individual pure compounds are beyond the scope of this work and are currently being addressed in an ongoing follow-up study. Within this defined scope, the findings aim to support the rational development of *O. corniculata* as a multi-target therapeutic candidate for diabetes mellitus and its associated cardiovascular complications.

## 2. Materials and Methods

### 2.1. Plant Extraction and Fractionation

Plant samples were collected from 3 locations of North Central region of Vietnam, namely Ba Vi (HN), Ninh Binh (NB), and Thanh Hoa (TH). After harvesting, botanical identification was performed based on comparative morphological analysis, and the scientific name was verified at the Vietnam National Museum of Nature by MSc. Bui Van Huong. The samples were identified as *Oxalis corniculata* L. (family Oxalidaceae), voucher specimen No. VNMN-040824 (HN), VNMN-100824 (NB), and VNMN-130824 (TH). Collected samples were thoroughly washed, dried at 40–45 °C to constant weight, and ground to a particle size of 1–3 mm prior to extraction and fractionation.

**Figure 1:**
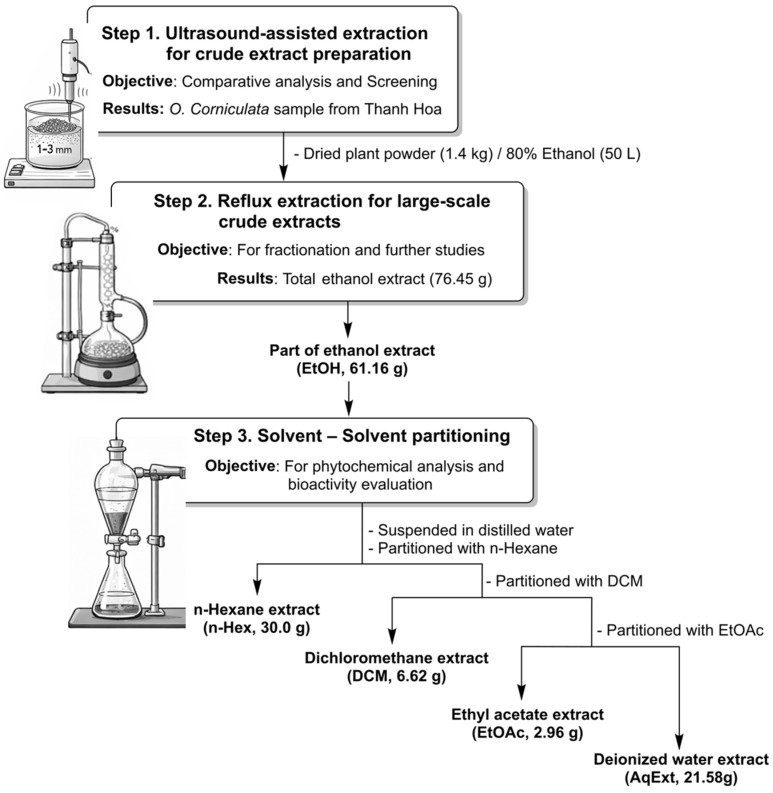
General workflow for extraction and fractionation of *O. corniculata* L. in different solvents.

In Step 1, as depicted in Figure 1, dried plant powder (1–3 mm) was subjected to ultrasound-assisted extraction (UAE). The material was extracted with solvent at a plant-to-solvent ratio of 1:10 (*v*/*w*) under ultrasonic irradiation at 55–60 °C for 2 h. The extract was filtered through cloth followed by Whatman filter paper. This procedure was repeated twice under identical conditions. The combined extracts were concentrated under reduced pressure (100 mbar, water bath at 60 °C) to constant weight of the crude extract.

Comparative chemical screening of crude extracts from different geographical origins was performed using thin-layer chromatography (TLC). TLC analysis was carried out on silica gel 60 F_254_ plates (Merck), pre-activated at 110 °C for 30 min. Samples were applied using a CAMAG Linomat 5 applicator with an application distance of 10 mm from the lower edge of the plate. Reference standards included luteolin, apigenin, and chlorogenic acid. Two mobile phase systems were employed: (I) toluene–ethyl acetate–formic acid (14:10:1, *v*/*v*/*v*) and (II) ethyl acetate–formic acid–acetic acid–water (100:11:11:26, *v*/*v*/*v*/*v*). After development, the plates were dried and visualized under UV light at 254 and 366 nm, followed by derivatization with NP/PEG reagent (naturstoff reagent A and polyethylene glycol) and re-examination at 366 nm.

In Steps 2 and 3, large-scale extraction and solvent–solvent partitioning were performed for fractionation. Dried plant powder (1.4 kg) was extracted by reflux with 80% ethanol (50 L). The extract was filtered and concentrated under reduced pressure to obtain the crude ethanol extract (EtOH). The dried EtOH extract was suspended in distilled water and successively partitioned with solvents of increasing polarity, including *n*-hexane (*n*-Hex), dichloromethane (DCM), and ethyl acetate (EtOAc). Each organic phase was collected and concentrated under reduced pressure to yield the corresponding fractions, namely *n*-hexane, dichloromethane, ethyl acetate, and the remaining aqueous fraction (AqExt).

### 2.2. Quality Control and Phytochemical Analysis

Qualitative phytochemical screening was performed as a quality control step prior to the determination of total phenolic and flavonoid contents. Standard chemical tests were applied to the crude extracts of the aerial parts of *O. corniculata* to assess major classes of secondary metabolites based on characteristic color reactions, precipitate formation, and fluorescence [11].

Alkaloids were examined using Mayer’s, Bouchardat’s, and Dragendorff’s reagents, while anthranoids were tested using 10% NaOH. Sterols were evaluated by the Liebermann–Burchard reaction, carotenoids by concentrated sulfuric acid treatment, and lipids by the filter paper test. Coumarins were screened using alkaline ring-opening/closing reactions, diazo coupling, and UV fluorescence. Flavonoids were assessed using ammonia vapor, ferric chloride complexation, and diazotization reactions. Cardiac glycosides were examined using Liebermann–Burchard, Legal, and Keller–Kiliani tests. Polysaccharides were evaluated with Lugol’s reagent, saponins by the froth test, tannins by gelatin, ferric chloride, and lead acetate reactions, and free reducing sugars, amino acids, and organic acids by Fehling’s, ninhydrin, and sodium carbonate tests, respectively.

#### 2.2.1. Determination of Total Phenolic Content (TPC)

The TPC of the total extract and fractions of the aerial parts of *O. corniculata* L. was determined using the Folin–Ciocalteu colorimetric method with minor modifications [12]. Briefly, 40 μL of each sample solution was mixed with 480 μL of diluted Folin–Ciocalteu reagent (1:10, *v*/*v*) and incubated at 40 °C for 1 min. Subsequently, 480 μL of sodium carbonate solution (6%, *w*/*v*) was added, and the reaction mixture was further incubated at 40 °C for 15 min. The absorbance was measured at 765 nm using a microplate reader. Gallic acid was used to construct the calibration curve, and TPC was expressed as milligrams of gallic acid equivalents per gram of extract (mg GAE/g extract).

#### 2.2.2. Determination of Total Flavonoid Content (TFC)

The TFC was determined using the aluminum chloride colorimetric method [12]. In brief, 240 μL of each sample solution was mixed with 40 μL of sodium nitrite solution (5%, *w*/*v*) and incubated at 25 °C for 6 min. Then, 40 μL of aluminum chloride solution (10%, *w*/*v*) was added, and the mixture was allowed to react for another 6 min. Subsequently, 400 μL of sodium hydroxide solution (1 M) and 280 μL of ethanol (30%, *v*/*v*) were added. The reaction mixture was incubated at room temperature for 15 min, and the absorbance was measured at 510 nm using a microplate reader. Quercetin was used as the reference standard, and TFC was expressed as milligrams of quercetin equivalents per gram of extract (mg QE/g extract).

### 2.3. α-Glucosidase Inhibition Activity Assay

The α-glucosidase inhibition assay was performed using a spectrophotometric method with some modifications [13]. *p*-nitrophenyl-α-D-glucopyranoside (pNPG) was used as a substrate. The α-glucosidase solution (1 U/mL) was prepared in 0.2 M potassium phosphate buffer (pH 6.8). Plant extracts and the positive control, acarbose, were dissolved and diluted in dimethyl sulfoxide (DMSO). Briefly, buffer (50 µL), enzyme (10 µL) and plant extracts at various concentrations (20 µL) were sequentially added to the wells, followed by incubation at 37 °C in the dark for 20 min. The reaction was initiated by adding 20 µL of pNPG solution (5 mM). After incubation for 30 min, 50 µL of Na_2_CO_3_ 0.1 M solution was added to stop reactions. Absorbance was measured at 405 nm using a microplate spectrophotometer. The percentage of inhibition (%I) was calculated as follows: %I = (OD_c_ − OD_s_)/OD_c_ in which OD_c_ and OD_s_ denote the absorbance of the control and sample, respectively. The half-maximal inhibitory concentration (IC_50_), defined as the concentration of extract required to inhibit 50% of α-glucosidase activity, was subsequently determined.

### 2.4. α-Amylase Inhibition Activity Assay

The inhibitory activity of the total extract and fractions of the aerial parts of *O. corniculata* on α-amylase was evaluated using a colorimetric method with minor modifications [14]. The extracts and the positive control acarbose were dissolved and diluted in DMSO. α-Amylase (10 U/mL) was prepared in phosphate buffer (pH = 6.9). Then, 50 µL of α-amylase solution (10 U/mL) was mixed with 50 µL of sample at various concentrations and pre-incubated at 37 °C for 10 min. The reaction was initiated by adding 50 µL of 1% (*w*/*v*) starch solution, followed by further incubation at 37 °C for 10 min. Subsequently, 100 µL of 3,5-dinitrosalicylic acid (DNS) reagent was added to terminate the reaction, and the mixture was heated at 100 °C for 10 min. After cooling to room temperature, 1 mL of distilled water was added. The absorbance was measured at 540 nm using a microplate reader. The percentage inhibition of α-amylase activity (%I) was calculated using the same equation described for the α-glucosidase assay, and the IC_50_ values were determined accordingly.

### 2.5. Antioxidant Activity Assays

#### 2.5.1. DPPH Free Radical Scavenging Assay

Antioxidant activities of the total extract and fractions of the aerial parts of *O. corniculata* L. were determined using the DPPH radical scavenging assay with ascorbic acid as the positive control, following the procedures described by Nguyen et al. [15]. Briefly, 20 μL of sample solutions at various concentrations were mixed with 180 μL of DPPH solution (0.1 mM in methanol) and incubated in the dark at room temperature for 20 min. The absorbance was measured at 517 nm. The percentage of radical scavenging activity and IC_50_ values were calculated using the same equation described previously.

#### 2.5.2. ABTS Radical Cation Decolorization Assay

The antioxidant capacity was further assessed using the ABTS radical cation decolorization assays with Trolox as the reference [15]. The ABTS radical was generated by reacting 7 mM ABTS with 2.45 mM potassium persulfate and allowing the mixture to stand in the dark at room temperature for 12–16 h. Prior to analysis, the ABTS solution was diluted to an absorbance of 0.70 ± 0.02 at 734 nm. Subsequently, 20 μL of sample solutions were mixed with 180 μL of ABTS solution and incubated in the dark for 6 min. The absorbance was recorded at 734 nm, and the IC_50_ values were determined using the same calculation method described above.

### 2.6. In Silico Screening of Bioactive Compounds

#### 2.6.1. Screening of Drug-like Compounds

Data on the chemical compounds present in the plant were collected through a literature survey of scientific articles available in databases such as PubMed, Google Scholar, and Web of Science. It is emphasized that the compounds used for in silico screening were collected from published phytochemical reports on *O. corniculata* and were not claimed to be exhaustively identified in the samples collected in this work. The compounds retrieved were standardized by name, and their SMILES and InChIKey codes were obtained from the PubChem database. Duplicate entries were removed, and unique identifiers were assigned for data management.

The collected compounds were further evaluated for oral bioavailability and drug-likeness using the Traditional Chinese Medicine Database and Analysis Platform (TCMSP) and SwissADME tools [16,17]. Compounds meeting at least one of the following criteria were considered to possess favorable drug-like properties with adequate oral bioavailability and were included in subsequent analyses:

- TCMSP indices: drug-likeness (DL) ≥ 0.18 and oral bioavailability (OB) ≥ 30%.
- SwissADME indices: good drug-likeness (≥2) and high gastrointestinal absorption (GI absorption: high).

#### 2.6.2. Molecular Docking Simulation

The chemical structures of the potential compounds were downloaded from the PubChem database and imported into MOE 2015.10 [18]. Partial atomic charges were assigned using the AM1-BCC charge model, and the ligand geometries were subsequently energy-minimized under the General Amber Force Field (GAFF) to obtain stable conformations prior to docking. The X-ray crystal structures of potential target proteins were retrieved from the Protein Data Bank (https://www.rcsb.org/). The docking simulations were performed in two steps with two groups of proteins: (i) structure-based screening of potential dual inhibitors from *O. corniculata* against α-glucosidase (PDB ID: 3TOP) [19] and α-Amylase (PDB ID: 4GQR) [20], and (ii) other hub protein targets participating in signaling pathways of type 2 diabetes and oxidative stress which had been identified through pharmacological network analysis described in the next section.

Protein structures were prepared using a standard protocol [21]. All crystallographic water molecules and nonessential co-crystallized components were removed. Binding sites were identified using MOE SiteFinder, with dummy atoms generated around the co-crystallized ligand [22]. Protonation states and atom types of key residues (His, Asn, Gln, and Pro) were optimized prior to docking [23]. To evaluate the docking compounds, the interactions and binding affinities were calculated using the London dG scoring function to give the first value, E_score1 (kcal/mol). The given poses were subsequently subjected for affinity dG refinement to estimate the binding energy E_score2 (kcal/mol). For each ligand, 100 poses were generated, and the 10 lowest-energy conformations were selected for analysis. The key ligand–protein interactions were visualized using Discovery Studio 2024 [24]. Co-crystallized ligands were used as references, and protocol validation was conducted by redocking, with the root mean square deviation (RMSD) and conserved interactions as evaluation criteria.

#### 2.6.3. Molecular Dynamics (MD) Simulation

Protein–ligand complexes with the most favorable docking scores were subjected to molecular dynamics (MD) simulations to assess complex stability and binding energetics. MD simulations were performed using GROMACS 2022 with the CHARMM36m force field, and system setup was generated via the CHARMM-GUI server [25,26]. Each complex was solvated in a rectangular water box with a 20 Å buffer and simulated under NPT conditions at 298.15 K for 100 ns. Structural stability and flexibility were evaluated using RMSD and RMSF analyses, respectively. Binding free energy was further estimated using the MM/PBSA approach [27].

### 2.7. Pharmacological Network for Exploring Antidiabetic and Antioxidant Mechanisms

#### 2.7.1. Prediction of Targets for Screened Compounds

**Figure 2:**
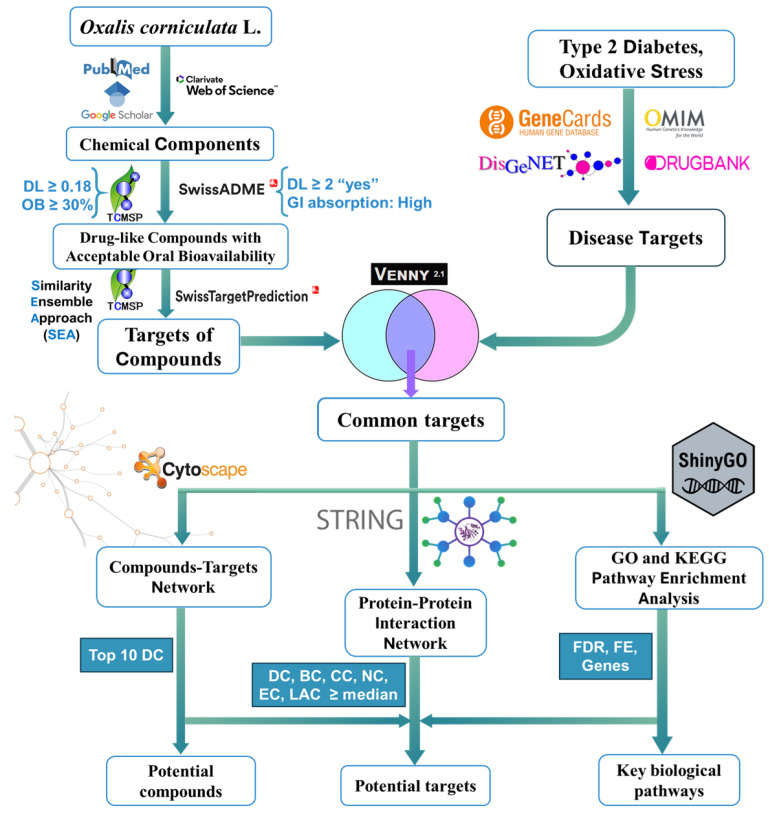
General workflow for exploring Anti-Type 2 Diabetes and Antioxidant mechanisms of *O. corniculata* L.

The orally drug-like compounds were input into three target prediction platforms: SwissTargetPrediction (http://swisstargetprediction.ch/), Similarity Ensemble Approach (SEA, https://sea.bkslab.org/), and the Traditional Chinese Medicine Systems Pharmacology Database (TCMSP, https://www.tcmsp-e.com/). Target prediction was restricted to Homo sapiens to ensure human relevance. For SwissTargetPrediction, only predicted targets with a non-zero probability score were retained, while SEA-derived targets were selected based on reported similarity confidence. TCMSP targets were retrieved according to the platform’s built-in reliability annotations. The predicted targets were retrieved in gene ID format and standardized using UniProtKB (https://www.uniprot.org/) (Figure 2).

#### 2.7.2. Collection of Target Information for Type 2 Diabetes and Oxidative Stress

Four databases were employed to collect information on disease-related targets: DrugBank (https://go.drugbank.com/), GeneCards (https://www.genecards.org/), DisGeNET (https://www.disgenet.org/search), and OMIM (https://omim.org/). For GeneCards, targets were filtered based on relevance score to exclude weak associations, while DisGeNET targets were selected according to curated evidence and confidence levels. The keywords used for the searches included “*type 2 diabetes*”, “*diabetic complications*”, “*oxidative*”, “*free radical*”, and “*reactive oxygen species*”. The databases were accessed between October 2024 to March 2025.

#### 2.7.3. Construction and Analysis of Compound–Target Interaction (CTI) Network

From the lists of compound-associated targets and disease-associated targets, common targets were identified using the Venny 2.1.0 tool. The interaction network between the screened compounds and the common targets was visualized with Cytoscape 3.9.1 [28]. The CytoNCA plugin was employed to analyze the topological parameters of the network [29]. Compounds and targets with higher degree values were identified as key and included for further analysis. The identification of common targets reflects the potential multi-target characteristics of phytochemicals and the complex pathophysiology of type 2 diabetes and oxidative stress.

#### 2.7.4. Construction and Analysis of Protein–Protein Interaction (PPI) Network

The gene IDs of the common targets were submitted to the STRING database to obtain the protein–protein interaction (PPI) network [30]. In this study, the confidence score was set to 0.7 (high confidence) to generate a network consisting of interactions that were most likely to occur in reality. The PPI data retrieved from STRING were imported into Cytoscape for visualization, and the CytoNCA plugin was applied to analyze the network topology. Six topological parameters were calculated, including: Degree centrality (DC), Betweenness centrality (BC), Closeness centrality (CC), Eigenvector centrality (EC), Network centrality (NC), and Local average connectivity (LAC) [31]. Proteins with parameter values greater than or equal to the median were considered hub proteins, representing key nodes in the network.

#### 2.7.5. GO and KEGG Pathway Enrichment Analysis

The list of common targets was subjected to enrichment analysis. This analysis was performed using two approaches: Gene Ontology (GO) and Kyoto Encyclopedia of Genes and Genomes (KEGG) pathways [32,33]. GO enrichment analysis was applied to interpret the data by identifying biological processes associated with the set of differentially expressed genes, while KEGG pathway analysis was used to determine the pathways in which the enriched genes were involved.

The gene IDs of the common targets were input into the ShinyGO 0.80 online tool (http://bioinformatics.sdstate.edu/go/) for both GO and KEGG enrichment analyses. The false discovery rate (FDR) threshold was set at ≤0.05. GO annotations, including molecular function (MF), cellular component (CC), and biological process (BP), as well as KEGG pathways most relevant to the input target list, were identified. The enriched terms were selected based on FDR values and ranked in descending order according to fold enrichment (FE). Overall, the network pharmacology analysis was used as a hypothesis-generating approach, and the predicted targets were further refined and validated through molecular docking and interaction analysis.

### 2.8. DFT Calculations

To investigate the non-enzymatic antioxidant mechanisms, selected potential compounds were subjected to DFT calculations to evaluate their antioxidant activity. The electronic properties of the compounds were calculated and compared with gallic acid as antioxidant reference. All calculations were performed using ORCA *v*.6.0 at the Becke three-parameter Lee–Yang–Parr (B3LYP) exchange–correlation functional level [34]. The 6−31+G(d,p) basis set was employed with B3LYP, as it provided an appropriate balance between computational cost and accuracy [35].

Geometry optimization and frequency calculations were carried out for the neutral forms (ArOH), the cationic species (ArOH^+^), and the corresponding radicals (ArO•) at the B3LYP/6−31+G(d,p) level. All calculations were performed in the gas phase at 298.15 K. The parameters used to assess antioxidant activity included: HOMO energy, LUMO energy, HOMO–LUMO energy gap, O–H bond dissociation enthalpy (BDE), and ionization potential (IP). BDE and IP were calculated using the following equations:

BDE = Δ*H_f_*(ArO•) + Δ*H_f_*(H•) − Δ*H_f_*(ArOH)

IP = Δ*H_f_*(ArOH^+^) − Δ*H_f_*(ArOH)

where Δ*H_f_*(ArOH+), Δ*H_f_*(ArOH), Δ*H_f_*(ArO•), and Δ*H_f_*(H•) represent the enthalpies of formation of the corresponding species.

## 3. Results and Discussion

### 3.1. TLC Analysis for Sample Selection

Samples of *O. corniculata* were collected from three locations in North Central Vietnam, including Ba Vi (HN), Ninh Binh (NB), and Thanh Hoa (TH), and taxonomically authenticated as *O. corniculata* Linn. (Oxalidaceae). Prior to extraction, TLC fingerprinting was conducted to compare the phytochemical profiles of the samples from different geographical origins.

**Figure 3:**
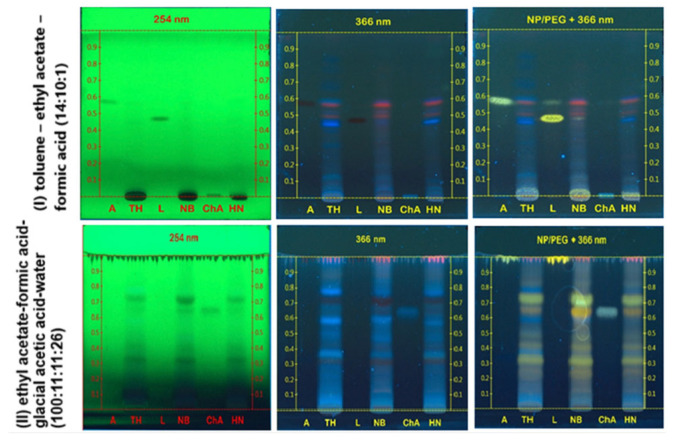
Chromatogram of *O. corniculata* samples developed with two mobile phases (I) and (II). Ba Vi (HN), Ninh Binh (NB) and Thanh Hoa (TH). Reference: A—apigenin, L—luteolin, ChA—chlorogenic acid. Plates were visualized under UV light at 254 nm and 366 nm, and after NP/PEG derivatization at 366 nm. Reference standards included apigenin (A), luteolin (L), and chlorogenic acid (ChA), exhibiting approximate Rf values of ~0.60–0.65, ~0.45–0.50, and ~0.25–0.30, respectively, in solvent system (II).

Figure 3 illustrates the TLC fingerprints of the samples developed using two mobile phases with different polarities. In both solvent systems, the chromatograms exhibited highly similar banding patterns with respect to Rf values and fluorescence behavior, indicating a comparable qualitative phytochemical composition among the samples. Under UV light at 366 nm following derivatization with NP/PEG reagent, several spots emitting yellow and blue fluorescence were consistently observed across all samples. These fluorescence characteristics are typical of flavonoids and phenolic acids [36]. Furthermore, partial co-migration with reference standards, including apigenin, luteolin, and chlorogenic acid, together with similar fluorescence behavior after NP/PEG derivatization, supported the possible presence of flavonoid and phenolic constituents in the *O. corniculata* extracts. However, it should be noted that Rf values and fluorescence characteristics alone are not sufficient for definitive compound identification, and the TLC analysis was primarily used for comparative fingerprinting and sample selection rather than structural confirmation.

**Figure 4:**
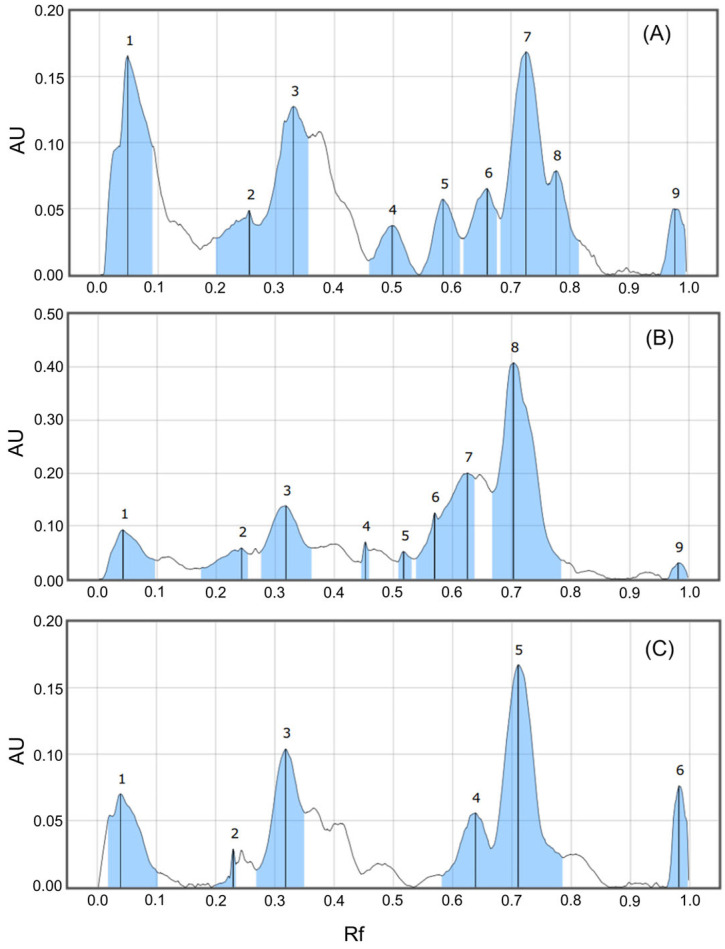
Densitometric profiles of *O. corniculata* samples developed with the mobile phase ethyl acetate–formic acid–glacial acetic acid–water (100:11:11:26), recorded at 366 nm after NP/PEG derivatization. Panels (**A**–**C**) correspond to TH, NB, and HN, respectively. The *y*-axis represents absorbance units (AU) obtained from TLC densitometric scanning, while the *x*-axis corresponds to the retention factor (Rf). Numbered peaks (1–9) denote major detected UV-active components and are used as reference markers for comparing relative phytochemical distribution patterns among samples rather than for definitive compound identification.

Among the three samples, the TH extract exhibited slightly higher spot intensity and density in both solvent systems, particularly in the medium-to-high Rf regions, suggesting a relatively higher abundance of UV-active secondary metabolites compared with the HN and NB samples. This observation was further confirmed by densitometric analysis of the chromatograms developed with solvent system (II) using visionCATS software v.3.0 (Figure 4). The densitometric profiles revealed highly comparable peak distribution patterns among the three samples over the Rf range of 0.05–0.80, confirming a stable and consistent phytochemical profile of *O. corniculata* across North Central region in Vietnam [37].

Several major peaks, particularly those located at Rf values around 0.30–0.35 and 0.65–0.75, were commonly observed in all samples. The chromatogram of the TH sample (Figure 3A) exhibited higher peak density and greater signal intensity at multiple Rf regions, especially those associated with medium- and high-polarity compounds, suggesting a relatively higher accumulation of phenolic and flavonoid constituents [38]. Thanh Hoa province is known for its favorable soil composition and climatic conditions, which support the growth of herbaceous medicinal plants such as *Centella asiatica* and *Oxalis* species [8,39]. Moreover, *O. corniculata* is widely distributed and traditionally used in traditional medicine in TH province. Therefore, TH sample was selected for subsequent extraction and fractionation.

### 3.2. Qualitative Phytochemical Analysis and Determination of TPC and TFC

Prior to quantitative analysis, a preliminary phytochemical screening of the total ethanol extract from the TH sample was performed to obtain an overview of its chemical composition. This screening was based on standard colorimetric, precipitation, and fluorescence-based chemical tests, as described in Section 2.2, which allow rapid identification of broad phytochemical groups rather than individual compounds.

The extract was found to contain flavonoids, coumarins, tannins, saponins, sterols, carotenoids, and lipids, while alkaloids, anthranoids, cardiac glycosides, polysaccharides, and reducing sugars were not detected. Flavonoids were consistently indicated by characteristic qualitative reactions, including ammonia vapor exposure, ferric chloride complexation, and diazotization tests, which are commonly used for the preliminary detection of flavonoid subclasses. The presence of tannins and coumarins, which are known for their antioxidant and enzyme-modulating activities, further suggested the biological potential of *O. corniculata*. These qualitative results are consistent with the TLC fingerprinting (Figure 3 and Figure 4) and provide a chemical basis for subsequent quantitative evaluation of total phenolic and flavonoid contents.

The TPC and TFC of the ethanolic extract and its solvent fractions from the aerial parts of *O. corniculata* are presented in Table 1. Notable differences in TPC and TFC were observed among the fractions, reflecting the selective extraction of phytochemical classes according to solvent polarity.

Among all fractions, the ethyl acetate fraction exhibited the highest levels of both TPC (85.00 mg GAE/g extract) and TFC (82.51 mg QE/g extract), indicating that the majority of phenolic and flavonoid constituents of *O. corniculata* are preferentially enriched in this semi-polar fraction. The aqueous fraction also showed relatively high TPC (67.62 mg GAE/g extract) and TFC (53.53 mg QE/g extract), suggesting that a considerable proportion of polar phenolics, particularly flavonoid glycosides, are extracted into water.

The crude ethanolic extract and the dichloromethane fraction displayed moderate levels of both TPC (65.52 and 50.26 mg GAE/g, respectively) and TFC (48.60 and 37.18 mg QE/g extract, respectively), consistent with their intermediate extraction polarity. In contrast, the *n*-hexane fraction showed the lowest values of both TPC (28.06 mg GAE/g extract) and TFC (24.81 mg QE/g extract), indicating that only a limited number of phenolic compounds, likely associated with lipophilic matrices, were present in this nonpolar fraction.

### 3.3. Inhibitory Effect on α-Glucosidase Enzyme

**Figure 5:**
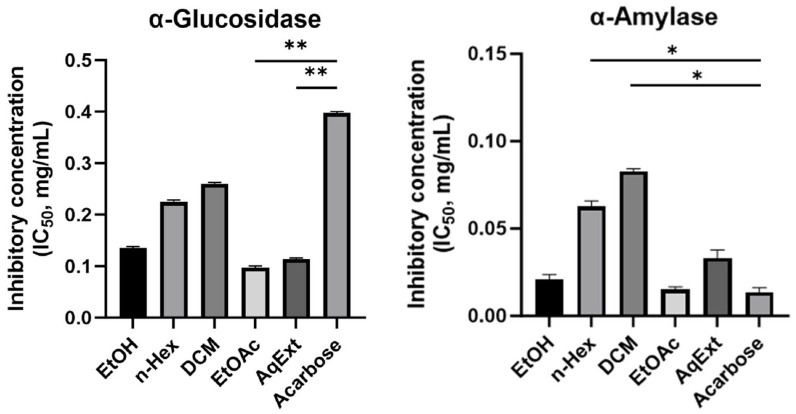
α-Glucosidase and α-amylase inhibitory activity (IC_50_, mg/mL) of *O. corniculata* extracts and fractions in different solvents. Data are presented as mean ± standard deviations (SD). Statistical comparisons were performed against the positive control acarbose using one-way ANOVA followed by Dunnett’s post hoc test; *p* < 0.05 (*) and *p* < 0.01 (**) indicate statistically significant differences.

The inhibitory effects of the ethanolic extract and solvent fractions of *O. corniculata* against α-glucosidase are summarized in Figure 5 and Supplementary Materials Table S1. All tested extracts and fractions exhibited measurable inhibitory activity, with IC_50_ values ranging from 0.097 to 0.260 mg/mL. Among them, EtOAc and AqExt fractions showed the strongest α-glucosidase inhibition, with an IC_50_ value of 0.097 mg/mL and 0.114 mg/mL, respectively, which was significantly lower than that of the positive control acarbose (0.397 mg/mL, *p* < 0.01).

The EtOH extracts exhibited moderate inhibitory activity, with IC_50_ values of 0.135 mg/mL, significantly stronger inhibition than acarbose (*p* < 0.05). In contrast, the *n*-hexane and DCM fractions displayed weaker activity (IC_50_ = 0.225 and 0.26 mg/mL). Overall, the α-glucosidase inhibitory activities of the extracts and fractions followed the descending order: EtOAc > AqExt > EtOH > *n*-Hex > DCM > acarbose.

### 3.4. Inhibitory Effect on α-Amylase Enzyme

The inhibitory activities of *O. corniculata* extracts against α-amylase are also presented in Figure 5 and Table S1. Compared to the α-glucosidase assay, the samples generally exhibited stronger inhibitory activity against α-amylase, as reflected by lower IC_50_ values, although greater variability among fractions was observed. The ethyl acetate fraction again showed the strongest α-amylase inhibition, with an IC_50_ value of 0.015 mg/mL, which was comparable to that of acarbose (0.013 mg/mL, *p* > 0.05).

The ethanolic extract demonstrated moderate α-amylase inhibitory activity (IC_50_ = 0.021 mg/mL), followed by the aqueous extract (IC_50_ = 0.032 mg/mL). In contrast, the *n*-hexane and DCM fractions exhibited weaker inhibitory effects, with IC_50_ values of 0.062 mg/mL and 0.081 mg/mL, respectively. Overall, the inhibitory potency against α-amylase decreased in the following order: EtOAc ≈ acarbose > EtOH > AqExt > *n*-Hex > DCM.

### 3.5. Antioxidant Activity Evaluated by DPPH and ABTS Assays

**Figure 6:**
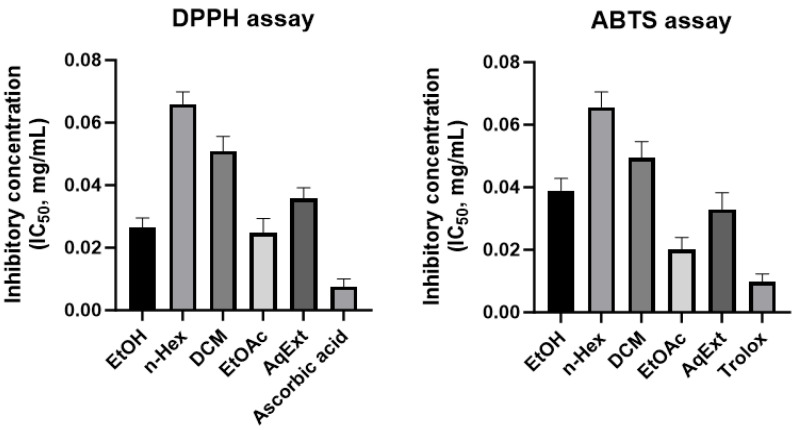
Evaluation of antioxidant activity of *O. corniculata* extracts and fractions against ABTS and DPPH radical species. Statistical analysis was performed using one-way ANOVA followed by Dunnett’s post hoc test, comparing each extract or fraction with the corresponding positive control (ascorbic acid for DPPH and Trolox for ABTS). Differences were considered statistically significant at *p* < 0.05.

The antioxidant activities of the ethanolic extract and solvent fractions of *O. corniculata* were evaluated using DPPH and ABTS radical scavenging assays, which are widely used chemical methods for assessing in vitro radical scavenging capacity rather than biological or cellular antioxidant effects. The results are summarized in Figure 6 and Table S2. In general, all extracts and fractions showed significantly weaker antioxidant effects than the corresponding positive controls (ascorbic acid for DPPH and Trolox for ABTS) (*p* < 0.05).

In the DPPH assay, the EtOAc fraction showed the strongest radical scavenging activity, with an IC_50_ value of 0.025 mg/mL, followed closely by the EtOH extract (0.027 mg/mL). AqExt displayed moderate activity (0.036 mg/mL), whereas the DCM and *n*-Hex fractions were less effective, with IC_50_ values of 0.051 and 0.066 mg/mL, respectively. As expected, the positive control ascorbic acid showed significantly stronger DPPH scavenging activity (0.008 mg/mL, *p* < 0.05).

A comparable trend was observed in the ABTS assay. The EtOAc fraction again demonstrated the highest antioxidant capacity (0.02 mg/mL), followed by the AqExt extract (0.033 mg/mL) and the EtOH extract (0.039 mg/mL). The DCM and *n*-Hex fractions showed weaker ABTS radical scavenging effects, with IC_50_ values of 0.049 mg/mL and 0.065 mg/mL, respectively. The reference standard Trolox, exhibited superior activity (0.01 mg/mL, *p* < 0.05). Overall, while all *O. corniculata* extracts and fractions demonstrated measurable chemical radical scavenging activity in both assays, their antioxidant capacities were consistently lower than those of the positive controls, following the descending order: EtOAc > EtOH ≈ AqExt > DCM > *n*-Hex.

### 3.6. In Silico Screening of Bioactive Compounds from O. corniculata

A comprehensive literature survey was conducted to compile the reported chemical constituents of *O. corniculata*. In total, 113 compounds were collected from phytochemical studies previously reported. The complete list of identified compounds is provided in Supplementary Table S3. For subsequent in silico screening, the chemical structures of all the compounds were retrieved from Pubchem database. These compounds were then evaluated for drug-likeness and oral bioavailability using established physicochemical and pharmacokinetic criteria [40]. As a result, 49 compounds satisfied the predefined screening thresholds and were selected for further investigation (Table 2). Each compound was assigned a molecular code (MOL01–MOL49) for consistency throughout the study.

The screened compounds belonged to diverse chemical classes (Table 2), including flavonoids, flavonoid glycosides, and isoflavonoids (32.7%), phenolic acids (benzoic and cinnamic acid derivatives, 18.4%), terpenoids (mono-, sesqui-, di-, tri-, sesterterpenoids, 12.2%), steroids/phytosterols (4.1%), fatty acids and fatty derivatives (8.2%), and other structures including lipids, amines, glycerophospholipids, ceramides, and heterocyclic compounds. Flavonoids constituted the largest group, with 15 compounds of the selected dataset. Other prominent groups included terpenoids and organic acids, while steroids, alkaloids, and other classes were represented to a lesser percentage [9].

#### 3.6.1. Molecular Docking Simulation with α-Glucosidase and α-Amylase

49 drug-like compounds were docked into the active sites of α-glucosidase and α-amylase, with 10 top-ranked poses per compound retained for statistical analysis. The binding affinities were evaluated at two hierarchical scoring levels [51,52]: (i) E_score1, derived from the London dG scoring function, and (ii) E_score2, calculated using the refined MOE affinity function after force-field optimization. Compounds were prioritized when they simultaneously exhibited low E_score1 values (strong initial recognition) and consistently low E_score2 values after refinement, together with good pose convergence (SD < 1.0 kcal/mol) across the retained poses. Based on this combined scoring strategy, ligands located in the lower-left region of the scatter plots, particularly those with E_score1 ≤ −12 kcal/mol for α-glucosidase and ≤−14 kcal/mol for α-amylase, and E_score2 values typically ≤−7 kcal/mol, were selected for further binding mode and interaction analyses.

**Figure 7:**
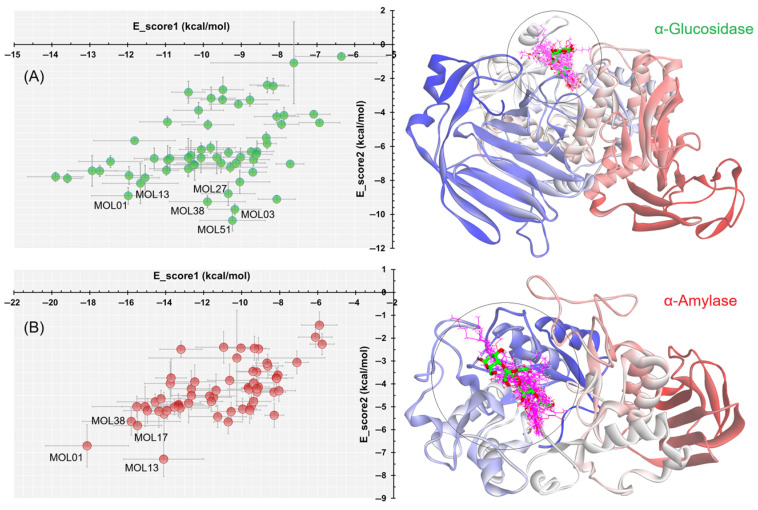
Distribution of binding energies (E_score1 and E_score2) for 49 drug-like compounds docked to (**A**) α-glucosidase (PDB ID: 3TOP) [19] and (**B**) α-amylase (PDB ID: 4GQR) [20]. Labeled points indicate representative ligands selected based on combined docking score magnitude and pose stability. Acarbose is displayed in C-green stick, whereas the docked natural compounds are shown in purple wire.

As illustrated in Figure 7, the majority of compounds are clustered within moderate-to-strong binding energy regions, with E_score1 values ranging from approximately −6 to −14 kcal/mol for α-glucosidase and −6 to −18 kcal/mol for α-amylase. SD values were generally low (mostly < 1.0 kcal/mol for E_score2), indicating good pose convergence and stable binding modes across the 10 retained conformations for most compounds.

For α-glucosidase (Figure 7A), several compounds exhibited strong and consistent binding, particularly those with E_score1 values below −12 kcal/mol and refined E_score2 values between −7 and −10 kcal/mol. Representative high-affinity ligands include MOL23, MOL36, MOL25, MOL28, and MOL40, which form a dense cluster in the lower-left region of the scatter plot. Some compounds (e.g., MOL03, MOL34, MOL38, MOL49) showed more favorable E_score2 than E_score1, suggesting that post-docking refinement enhanced key interactions, such as hydrogen bonding and π–π stacking, once the ligand–protein complex was energetically relaxed.

In contrast, docking against α-amylase (Figure 7B) yielded more negative E_score1 values overall, with several compounds reaching −15 to −18 kcal/mol at the initial scoring stage (e.g., MOL01, MOL08, MOL13, MOL38, MOL40). This suggests a larger and more accommodating binding cavity, capable of stabilizing bulky or flexible ligands. However, after refinement, E_score2 values converged to a narrower range (approximately −4 to −6 kcal/mol), indicating that some initially strong London scores may reflect non-specific hydrophobic contacts that were partially penalized during force-field optimization. Despite this reduction, several compounds (e.g., MOL01, MOL13, and MOL17) maintained consistently favorable E_score2 values with low SDs, supporting their potential as dual inhibitors of both α-glucosidase and α-amylase.

**Figure 8:**
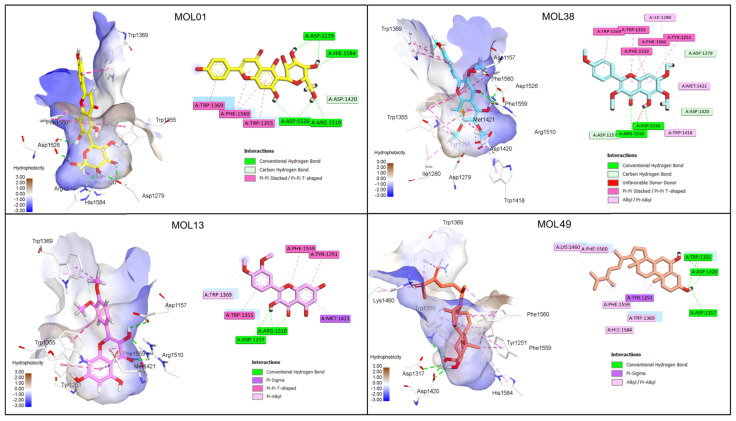
Molecular docking poses and key intermolecular interactions of the top-ranked compounds in the catalytic pocket of α-glucosidase (PDB ID: 3TOP) [19].

Based on the docking results, four compounds, namely MOL01, MOL13, MOL38, and MOL49, were selected for detailed interaction analysis with α-glucosidase. Figure 8 illustrates the corresponding 2D and 3D interaction patterns of these compounds within the active site of the enzyme. α-Glucosidase is a key digestive enzyme belonging to the glycoside hydrolase family, characterized by a (β/α)\_8_ TIM-barrel fold that forms a deep and well-defined catalytic pocket [19,53]. The active site is composed of several conserved residues involved in substrate recognition and catalysis, especially Asp1279, Asp1420, Glu1534, His1584, Arg1510, Phe1560, Trp1369, and Trp1355, which have been consistently reported to play crucial roles in hydrogen bonding, π–π stacking, and stabilization of transition states during glycosidic bond cleavage [19].

Among the selected compounds, MOL49 exhibited the strongest refined binding affinity, with an E_score2 of −10.36 kcal/mol, indicating a highly stable binding mode after force-field refinement. This compound penetrates deeply into the catalytic cavity and forms multiple hydrogen bonds with Asp1279 and Asp1420, while additional hydrophobic and alkyl interactions with Phe1560 and His1584 further stabilize the complex. Meanwhile, the two flavonoids MOL01 and MOL13 also demonstrated strong binding profiles by forming extensive hydrogen-bond networks involving Asp1279, Glu1534, and Arg1510, together with π–π stacking interactions with Trp1369 and Phe1560, which contribute to anchoring the ligands within the active site. MOL38, despite exhibiting a moderately less favorable E_score1, showed a markedly improved refined binding affinity with an E_score2 of −9.25 kcal/mol.

**Figure 9:**
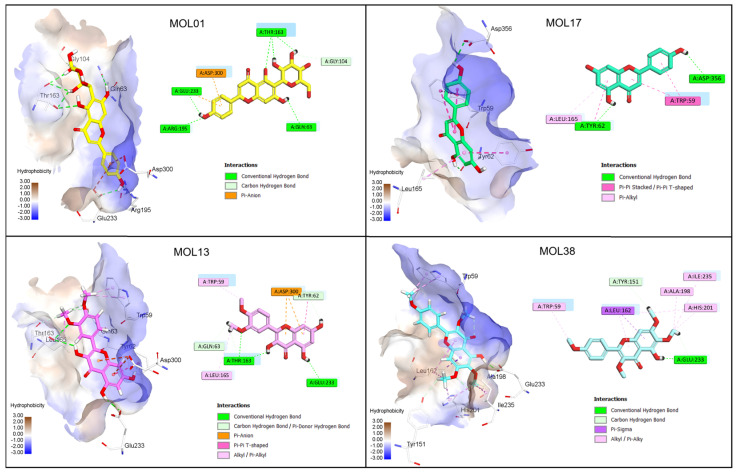
Molecular docking poses and key intermolecular interactions of the top-ranked compounds in the catalytic pocket of α-amylase (PDB ID: 4GQR) [20].

On the other hand, four compounds (MOL01, MOL13, MOL17, and MOL38) were selected for detailed interaction analysis with α-amylase. As shown in Figure 9, all selected ligands bind within the catalytic cleft of α-amylase, a glycoside hydrolase family 13 enzyme characterized by a (β/α)\_8_ TIM-barrel fold [20]. The active site is defined by the conserved catalytic triad Asp197, Glu233, and Asp300, together with aromatic residues such as Trp59 and Trp58, which are known to play key roles in substrate recognition and stabilization through hydrogen bonding and π–π interactions [20].

Among the selected compounds, MOL01 showed the most favorable overall docking performance, with a strong initial docking score (E_score1 = −18.14 kcal/mol) and a refined binding affinity of E_score2 = −6.71 kcal/mol. This high affinity is supported by an extensive hydrogen-bonding network involving the catalytic residues Asp197 and Glu233, together with additional interactions with Asp300 and His299, indicating a well-anchored and stable binding mode. MOL13 and MOL38 also exhibited strong docking profiles, with E_score1 values of −14.11 and −15.81 kcal/mol, respectively, and refined affinities in the range of −7.29 kcal/mol (MOL13) and −5.64 kcal/mol (MOL38). Their binding modes are dominated by hydrophobic and π–π stacking interactions with Trp59 and Trp58, suggesting effective blockage of the substrate-binding region rather than direct catalytic residue engagement. MOL17 displayed a balanced interaction profile with the formation of a salt bridge with Asp197 and hydrogen bonds with both Glu233 and Asp300. Its elongated structure enables extensive van der Waals contacts across the binding pocket, contributing to its stable binding conformation after refinement.

#### 3.6.2. Molecular Dynamics Simulation Analysis and MM/PBSA Calculations

**Figure 10:**
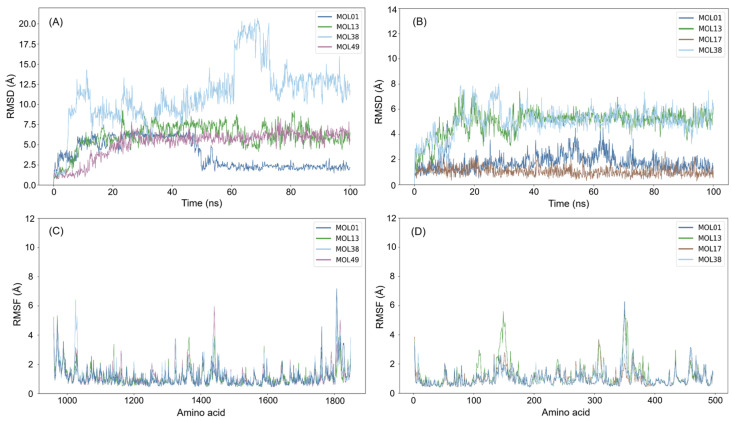
RMSD and RMSF analyses from 100 ns molecular dynamics simulations of ligand complexes with α-glucosidase (**A**,**C**) and α-amylase (**B**,**D**).

To validate the docking results, 100 ns molecular dynamics simulations were conducted for selected high-affinity compounds (MOL01, MOL13, MOL17, MOL38, and MOL49) in complex with α-glucosidase and α-amylase, using acarbose as the reference. RMSD analyses showed that all complexes reached stable equilibrated states after an initial relaxation phase, supporting the reliability of the docked binding poses. α-Glucosidase complexes displayed higher RMSD values (~5–12 Å) than those of α-amylase (~6–8 Å), reflecting the greater intrinsic flexibility of α-glucosidase (Figure 10).

RMSF analysis revealed higher flexibility in α-glucosidase, with pronounced fluctuations (>8 Å) in loop or terminal regions (~1200–1800 residues), while catalytic core residues remained relatively rigid (~2–4 Å). In contrast, α-amylase complexes showed lower overall fluctuations (~2–8 Å) restricted to fewer regions (~100–400 residues), indicating a more structurally constrained binding pocket. As can be seen in Figure 8, catalytic residues in both enzymes exhibited minimal fluctuations, suggesting that ligand binding preserves active-site integrity.

Binding free energy calculations using the MM/PBSA method provided quantitative insight into ligand affinity (Table 3). Consistent with docking energies, MM/PBSA calculations revealed that MOL01 and MOL49 bind strongly to α-glucosidase (−102.45 and −94.05 kJ/mol, respectively), approaching the affinity of acarbose (−114.12 kJ/mol), while MOL13 and MOL01 showed the most favorable binding to α-amylase (−93.24 and −85.01 kJ/mol). Overall, the combined docking, RMSD/RMSF, and MM/PBSA results confirm the stability of the protein–ligand complexes and highlight MOL01 as a promising dual inhibitor, with MOL13 and MOL49 showing target-selective potential.

### 3.7. Polypharmacological Network to Explore Multi-Targeted Mechanisms of O. corniculata

Beyond the dual inhibitory effects on α-glucosidase and α-amylase, this study further explored the multi-targeted mechanisms underlying the antidiabetic and antioxidant activities of *O. corniculata* aerial parts using a network pharmacology approach. This strategy allows a system-level understanding of how multiple bioactive compounds simultaneously regulate diverse molecular targets involved in Type 2 Diabetes Mellitus (T2DM) and oxidative stress.

#### 3.7.1. Antidiabetic and Antioxidant Target Prediction

To elucidate the multi-targeted mechanisms underlying the antidiabetic and antioxidant activities of *O. corniculata*, the potential molecular targets of 49 drug-like compounds were predicted using SwissTargetPrediction, SEA, and TCMSP, as described in the Section 2.7.2. Both experimentally validated and computationally predicted targets were included. After gene name standardization and removal of duplicates, a total of 1464 unique compound-related targets were obtained. In parallel, disease-associated genes related to T2DM and oxidative stress were collected from DrugBank, GeneCards, DisGeNET, and OMIM databases, yielding 3721 T2DM-related genes and 2803 oxidative stress–related genes.

**Figure 11:**
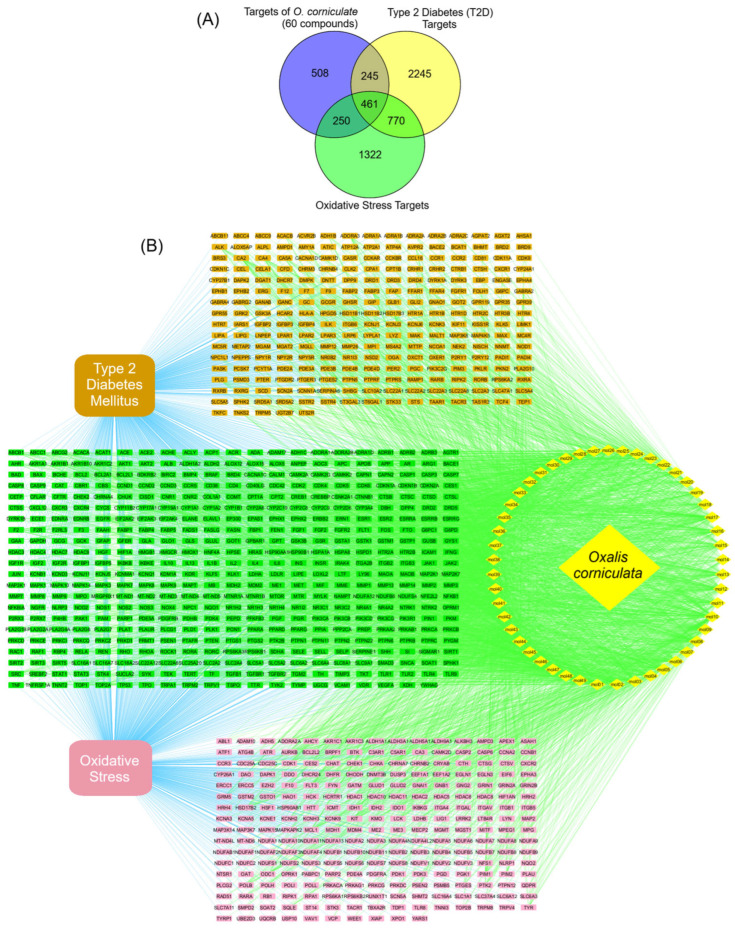
(**A**) Venn diagram and (**B**) compound–target interaction network illustrating the relationships between *O. corniculata*, type 2 diabetes mellitus (T2DM), and oxidative stress. Node colors indicate shared targets: *O. corniculata*–T2DM (mustard yellow), *O. corniculata*–oxidative stress (purple), and *O. corniculata*–T2DM–oxidative stress (green).

Venn diagram analysis was subsequently performed to identify overlapping targets between compound-related targets and disease-associated genes. The results revealed 706 common targets between *O. corniculata* compounds and T2DM, 711 targets shared with oxidative stress, and 461 targets overlapping both pathological conditions (Figure 11A). After merging these datasets and eliminating redundancies, a total of 956 common targets were retained for further compound–target network construction and downstream network pharmacology analyses (Supplementary Table S4).

This target prediction strategy was intentionally inclusive to capture the broad chemical space and potential polypharmacological nature of *O. corniculata* and should therefore be interpreted as hypothesis-generating rather than as evidence of direct target engagement. Only targets annotated for Homo sapiens were retained for downstream analyses to enhance biological relevance, and confidence thresholds followed the default or recommended settings of each platform, as detailed in Section 2.7.

#### 3.7.2. Protein–Protein Interaction Network (PPIN) Analysis

**Figure 12:**
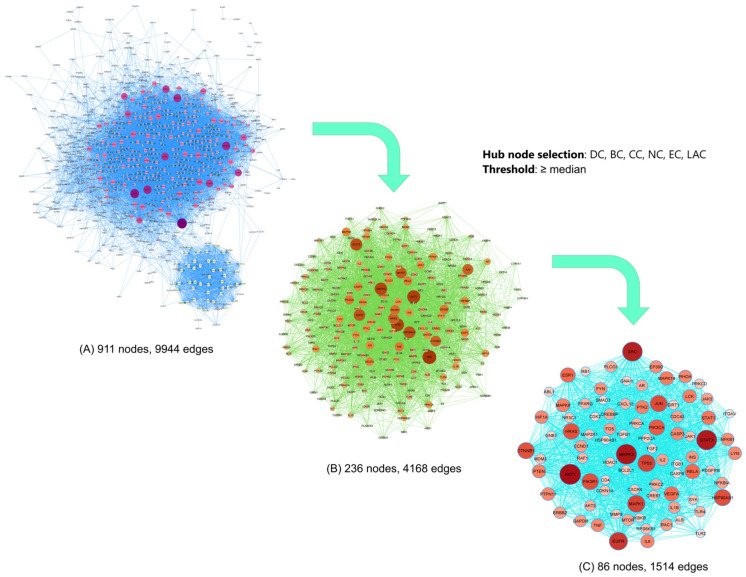
Two-step topological filtering analysis of the PPIN to identify core targets of *O. corniculata*. The size and color intensity of the nodes correspond to their DC values. Hub nodes were filtered based on six parameters, including degree centrality (DC), betweenness centrality (BC), closeness centrality (CC), neighborhood connectivity (NC), eigenvector centrality (EC), local average connectivity (LAC), with median value as the threshold.

To elucidate the functional relationships among the shared targets and to identify key proteins involved in the multi-target mechanisms of *O. corniculata*, a protein–protein interaction network (PPIN) was constructed based on the 956 common targets associated with T2DM and oxidative stress. The PPIN was generated using the STRING database, including only high-confidence interactions (interaction score ≥ 0.7). The resulting network consisted of 911 nodes and 9944 edges, indicating extensive functional connectivity among proteins involved in metabolic regulation and redox homeostasis (Figure 12).

Topological analysis of the PPIN was performed using six centrality parameters (DC, BC, CC, EC, NC, and LAC) [31]. Proteins with values above the median for all parameters were retained through two successive filtering steps, yielding a core network of 86 hub proteins with 1514 interactions (Supplementary Table S5). Key nodes such as AKT1, MGAM, EGFR, TNF, IL6, and MAPK14 were identified, highlighting pathways related to insulin signaling, inflammation, and oxidative stress.

It should be noted that several of these hub proteins (e.g., AKT1, TNF, IL6, MAPKs) represent central signaling nodes that are broadly involved in metabolic and inflammatory diseases, and their identification here reflects network centrality rather than pathway specificity. These findings suggest that *O. corniculata* may potentially exert its antidiabetic and antioxidant effects through coordinated modulation of metabolic–inflammatory–oxidative networks, consistent with its polypharmacological nature.

#### 3.7.3. GO Enrichment Analysis and KEGG Signaling Pathways

**Figure 13:**
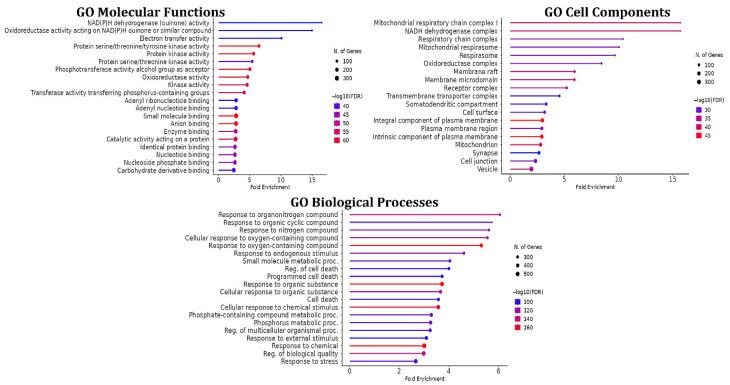
GO enrichment analysis according to MF, CC and BP categories.

GO enrichment analysis was performed on the 956 common targets using ShinyGO *v*0.80 to characterize their functional roles at the molecular, cellular, and biological levels (Figure 13). In the molecular function (MF) category, the most significantly enriched terms included carbohydrate binding (GO:0097367), small molecule binding (GO:0036094), oxidoreductase activity (GO:0016491), NAD(P)H dehydrogenase activity (GO:0003955), and electron transfer activity (GO:0009055), suggesting that the targets are closely associated with glucose metabolism and cellular redox regulation.

For cellular component (CC), enriched terms were mainly related to metabolically active and signaling-associated cellular compartments, including mitochondrial and cytosolic components, supporting the involvement of these targets in energy metabolism and oxidative stress responses. In the biological process (BP) category, dominant terms included response to organic substances (GO:0010243), response to chemical stimulus (GO:0070887), small-molecule metabolic process (GO:0044281), response to endogenous stimulus (GO:0009719), response to oxidative stress (GO:0006979), and regulation of apoptotic process (GO:0008219), all of which are strongly implicated in the pathogenesis of T2DM and oxidative damage.

KEGG pathway enrichment analysis further revealed that the common targets are involved in multiple signaling pathways related to metabolic dysregulation, inflammation, and oxidative stress [33]. Interestingly, pathways directly associated with T2DM, including the AGE–RAGE signaling pathway in diabetic complications (hsa04933), insulin resistance (hsa04931), diabetic cardiomyopathy (hsa05415), and metabolic pathways (hsa01100), were among the most significantly enriched with extremely low FDR values. The AGE–RAGE pathway contained the highest number of mapped targets, including key inflammatory and oxidative stress–related proteins such as TNF, IL6, and MAPK14, highlighting its central role in mediating chronic inflammation, ROS generation, and diabetic complications [54]. Detailed KEGG pathway analysis could be found in Supplementary Materials (Supplementary Figures S1–S5). Importantly, the enrichment of these pathways indicates statistical association at the network level and does not imply direct activation or inhibition of the corresponding signaling cascades in biological systems.

In addition, several oxidative stress– and inflammation-related pathways, such as chemical carcinogenesis—reactive oxygen species (hsa05208), MAPK signaling pathway (hsa04010), FoxO signaling pathway (hsa04068), cAMP signaling pathway (hsa04024), and calcium signaling pathway (hsa04020), were significantly enriched. The involvement of targets in these pathways indicates that *O. corniculata* may exert its antidiabetic effects not only through direct glycemic control but also via modulation of oxidative stress, inflammatory signaling, and cell survival pathways, supporting a polypharmacological and systems-level mechanism of action.

#### 3.7.4. Target Validation by Molecular Docking Simulations

Based on PPIN and GO/KEGG enrichment analyses, several core targets beyond α-glucosidase and α-amylase were identified, including AKT1, EGFR, TNF-α, IL6, MAPK14 (p38 MAPK), PTGS2 (COX-2), PTPN1 (PTP1B), and xanthine oxidase (XO). These proteins are centrally involved in insulin signaling, inflammation, and oxidative stress.

**Figure 14:**
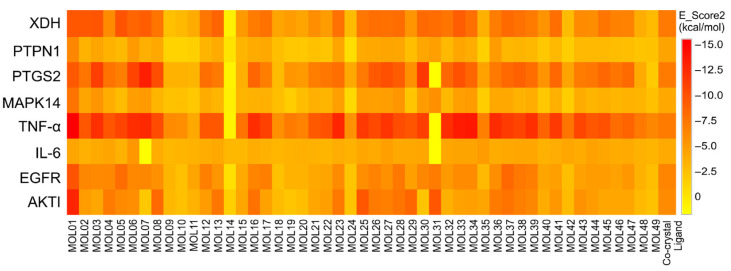
The heat map showing the binding energy of 49 compounds of *O. corniculata* with 8 potential targets beyond α-glucosidase and α-amylase. RAC-alpha serine/threonine-protein kinase (AKT1, PDB ID: 4GV1) [55], epidermal growth factor receptor (EGFR, PDB ID: 1M17) [56], interleukin-6 (IL6, PDB ID: 1ALU) [57], Tumor necrosis factor alpha (TNFα, PDB ID: 2AZ5) [58], p38 MAP kinase (MAPK14, PDB ID: 1A9U) [59], COX-2 (PTGS2, PDB ID: 1PXX) [60], protein-tyrosine phosphatase 1B (PTPN1, PDB ID: 1NNY) [61], and xanthine oxidase (XDH, PDB ID: 3NVY) [62].

Molecular docking was performed to validate the network-based predictions. Docking score analysis showed that most compounds exhibited favorable binding energies, with particularly strong affinities toward TNF-α, xanthine oxidase (XO), and COX-2, underscoring their relevance to the anti-inflammatory and antioxidant effects of *O. corniculate* (Figure 14). Several compounds, including MOL01, MOL49, MOL03, MOL08, and MOL05, demonstrated strong binding affinities across multiple targets, suggesting a multi-target (polypharmacological) interaction pattern. Among them, isovitexin (MOL01) showed the most prominent binding performance, with an average docking score of −10.06 kcal/mol across all investigated targets, corresponding to a strong binding affinity.

The heatmap analysis revealed a clear binding preference toward TNF-α, followed by xanthine oxidase (XO) and COX-2 (PTGS2), highlighting these proteins as key molecular targets potentially underlying the antidiabetic, anti-inflammatory, and antioxidant activities of the plant. The top 10 compounds with the highest binding affinities for these four targets are summarized in Table 4. This docking-based ranking was highly consistent with the top candidate compounds predicted from the compound–target interaction network, thereby confirming the reliability of the network pharmacology predictions and supporting the relevance of the selected molecular targets.

### 3.8. DFT Calculations to Evaluate Radical Scavenging Activity

Given the well-established antioxidant potential of flavonoids and their suitability for quantum chemical calculations, DFT analysis was performed on six flavonoid candidates identified from the compound–target interaction network, using gallic acid as a reference. All selected compounds belonged to the flavone subclass and were chosen to rationalize structure–antioxidant activity trends at the molecular level.

**Figure 15:**
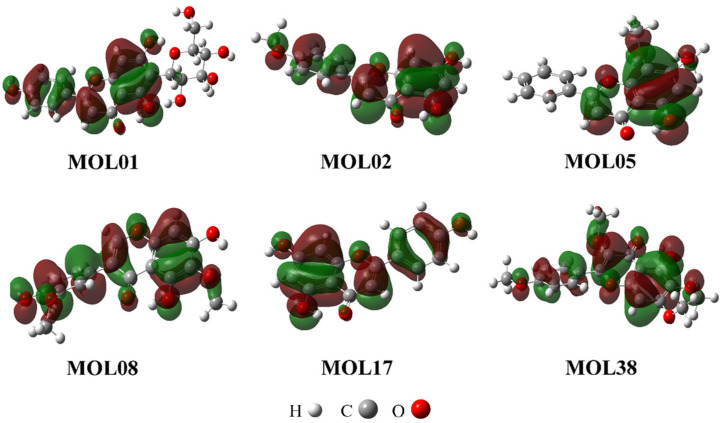
HOMO orbitals of six compounds investigated by DFT. Green and red isosurfaces indicate positive and negative phases of the HOMO, respectively.

As summarized in Table 5, the flavonoids exhibited HOMO energy levels (−5.96 to −6.44 eV) comparable to gallic acid (−6.52 eV), while showing consistently smaller HOMO–LUMO gaps (3.86–4.14 eV) than of gallic acid (4.93 eV). Cartesian coordinates of all the structures optimized using DFT calculations were provided in Supplementary Materials Tables S6–S12. Reduced energy gaps indicate enhanced electronic reactivity and a greater propensity to participate in radical neutralization processes. HOMO electron density maps (Figure 15) revealed electron delocalization primarily over the flavone aglycone, supporting the role of the conjugated π-system in antioxidant activity [63].

It should be noted that all DFT calculations were conducted in the gas phase, and therefore the resulting electronic descriptors reflect intrinsic molecular properties rather than solvent- or biology-specific effects. Accordingly, these parameters are intended to provide mechanistic insights into radical scavenging tendencies rather than direct quantitative predictors of biological antioxidant potency.

BDE and IP calculations (Table 6) provided mechanistic insight into the antioxidant properties of the selected flavonoids. In the embedded structural diagram, capital letters denote the aromatic and heterocyclic rings, numbers indicate carbon positions, and red labels mark the hydroxyl groups selected for BDE evaluation. The O–H BDE of the 4-OH group of gallic acid was used as a reference due to its strong reducing capacity and ability to form a stable phenoxyl radical [63]. According to the hydrogen atom transfer (HAT) mechanism, lower BDE values indicate a greater propensity for hydrogen donation. All investigated flavonoids exhibited BDE values comparable to or lower than gallic acid (92.63 kcal/mol), suggesting favorable radical-scavenging activity. In particular, MOL08 showed the lowest BDE (74.39 kcal/mol), followed by MOL01, MOL17, and MOL02, indicating superior HAT-based antioxidant potential.

A position-specific analysis revealed that, in MOL17, MOL01, and MOL08, the 4′-OH group on the B ring consistently exhibited the lowest BDE values, making it the most favorable site for hydrogen abstraction compared with the 5-OH and 7-OH groups on the A ring. This trend can be explained by enhanced electron delocalization arising from the C2=C3 double bond and the C4 carbonyl group, which stabilizes the phenoxyl radical. Higher BDE values at the 5-OH position are likely due to intramolecular hydrogen bonding with the 4-oxo group. Compared with its aglycone apigenin, isovitexin (M01) showed slightly lower BDE values at the 4′-OH and 5-OH positions, whereas the BDE at 7-OH was marginally higher, possibly due to hydrogen bonding between the 7-OH group and the glucose moiety [64].

In addition, IP values supported the single-electron transfer (SET) mechanism. All flavonoids exhibited lower IP values than gallic acid (182.62 kcal/mol), indicating enhanced electron-donating ability [65]. Consistent with the BDE results, MOL08 displayed the lowest IP value (163.09 kcal/mol), highlighting its strong antioxidant potential via both HAT and SET pathways. Overall, these DFT findings corroborate previous data for apigenin and extend antioxidant mechanistic insights to less-studied flavonoids from *O. corniculata*.

## 4. Conclusions

This study provided a comprehensive study of *O. corniculata* aerial parts collected from North Central Vietnam as a potent natural inhibitor of α-glucosidase and α-amylase. Among the extracts and fractions investigated, the ethyl acetate fraction, characterized by the highest flavonoid content, exhibited the highest potential dual-enzyme inhibitory activity, outperforming other fractions while simultaneously demonstrating strong antioxidant capacity in DPPH and ABTS assays.

Beyond digestive enzyme inhibition, the integration of multi-scale in silico approaches revealed a broader and more complex antidiabetic and antioxidant mechanism. Molecular docking, molecular dynamics simulations, and MM/PBSA calculations identified isovitexin as a stable and energetically favorable dual inhibitor of α-glucosidase and α-amylase, while additional flavonoids contributed to enzyme selectivity and target diversity. Network pharmacology analysis further demonstrated that *O. corniculata* exerts polypharmacological effects by modulating key signaling pathways related to insulin resistance, inflammation, and oxidative stress, including AKT1, MAPK, TNF-α, and IL6. Complementary DFT calculations elucidated the molecular basis of antioxidant activity, highlighting low bond dissociation enthalpies and ionization potentials that support efficient radical scavenging via HAT and SET mechanisms. Overall, this integrated experimental–computational framework positions *O. corniculata* as a scientifically substantiated, multi-target phytotherapeutic candidate for diabetes management, extending its therapeutic relevance well beyond α-glucosidase and α-amylase inhibition.

Despite the comprehensive integration of in vitro assays and multi-scale in silico analyses, the present study has several limitations. First, the chemical characterization of the ethyl acetate fraction was based on TLC fingerprinting and literature data, and comprehensive chemical profiling was not yet included. Second, the biological activities were evaluated at the fraction level, while individual compounds such as isovitexin were not yet isolated and experimentally validated in vitro. These aspects are currently being addressed in our ongoing follow-up study, which focuses on HPLC-QToF-MS-guided phytochemical profiling, compound isolation, structural confirmation, and direct enzyme inhibition and antioxidant assays of purified constituents.

## Figures and Tables

**Table 1:** TPC and TFC of aerial *O. corniculata* extract and fractions.

Extract and Fractions	TPC (mg GAE/g Extract)	TFC (mg QE/g Extract)
EtOH	65.52 ± 2.72	48.60 ± 0.47
*n*-Hex	28.06 ± 1.92	24.81 ± 4.39
DCM	50.26 ± 3.97	37.18 ± 1.12
EtOAc	85.00 ± 1.61	82.51 ± 0.85
AqExt	67.62 ± 0.71	53.53 ± 0.93

**Table 2:** 49 compounds from *O. corniculata* with drug-like properties and acceptable oral bioavailability.

Cpd. ID	Name	Chemical Class	References
MOL01	Isovitexin	Flavonoid glycoside	[41,42]
MOL02	Acacetin	Flavonoid	[41,42]
MOL03	β-Sitosterol	Steroid	[42,43]
MOL04	Embelin	Benzoquinone	[44]
MOL05	5-Hydroxy-7,8-dimethoxyflavone	Flavonoid	[41,42]
MOL06	Eburicoic acid	Triterpenoid	[45]
MOL07	Squalene	Triterpenoid	[46]
MOL08	Iristectorigenin A	Isoflavonoid	[47]
MOL09	*p*-Hydroxybenzoic acid	Benzoic acid	[42,43,48]
MOL10	Vanillic acid	Benzoic acid	[42]
MOL11	Syringic acid	Benzoic acid	[42]
MOL12	Apigenin 7,4′-dimethyl ether	Flavonoid	[41]
MOL13	Dillenetin	Flavonoid	[41]
MOL14	Oxalic acid	Carboxylic acid	[42]
MOL15	Malic acid	Hydroxy acid	[8]
MOL16	3,5,2′-Trihydroxy-7,5′-dimethoxyflavone	Flavonoid	[42,43]
MOL17	Apigenin	Flavonoid	[42]
MOL18	Gallic acid	Benzoic acid	[42]
MOL19	Harmine	Alkaloid	[42]
MOL20	Harmaline	Alkaloid	[42]
MOL21	Ascorbic acid	Furanone	[8]
MOL22	Parthenin	Sesquiterpene lactone	[44]
MOL23	Petunidin	Flavonoid	[44]
MOL24	Octanal	Aldehyde	[44]
MOL25	Lecanoric acid	Phenylpropanoid	[44]
MOL26	Dihydrosphingosine	Amine	[44]
MOL27	Ophiobolin A	Sesterterpenoid	[44]
MOL28	Phytosphingosine	Amine	[44]
MOL29	Pyruvic acid	Keto acid	[49]
MOL30	N-acetylsphinganine	Ceramide	[44]
MOL31	1-Linolenoyl lysolecithin	Glycerophospholipid	[44]
MOL32	2-Pentylfuran	Furan	[49]
MOL33	Rotenone	Isoflavonoid	[49]
MOL34	(+)-Larixol	Diterpenoid	[44]
MOL35	Sebacic acid	Fatty acid	[44]
MOL36	Malvidin	Flavonoid	[44]
MOL37	5-O-Desmethylnobiletin	Carbohydrate	[41,42]
MOL38	5-hydroxy-3,6,7,4′-tetramethoxyflavone	Flavonoid	[41,42]
MOL39	Penduletin	Flavonoid	[41,42]
MOL40	Irigenin	Isoflavonoid	[47]
MOL41	Linalyl anthranilate	Monoterpenoid	[50]
MOL42	Tectorigenin	Isoflavonoid	[47]
MOL43	Caffeic acid	Cinnamic acid	[48]
MOL44	Palmitic acid	Fatty acid	[46,50]
MOL45	*p*-Coumaric acid	Cinnamic acid	[48]
MOL46	*E*-14-Hexadecenal	Aldehyde	[50]
MOL47	*E*-15-Heptadecenal	Aldehyde	[50]
MOL48	Sinapic acid	Cinnamic acid	[48]
MOL49	24-Methylenecholest-4-ene-3*β*,6*β*-diol	Steroid	[45]

**Table 3:** MM/PBSA binding free energies (kJ/mol) of potential compounds from MD simulations.

Compound	MM/PBSA Binding Free Energy (kJ/mol)	
	α-Glucosidase	α-Amylase
MOL01	−102.45	−85.01
MOL13	−78.47	−93.24
MOL17	ND *	−68.52
MOL38	−52.13	−64.64
MOL49	−94.05	ND
Acarbose	−114.12	−107.98

* ND: Non determined.

**Table 4:** Top 10 compounds with the highest binding affinities toward TNF-α, XO, and COX-2.

TNF-α		XO		COX-2	
Compounds	E_Score2 (kcal/mol)	Compounds	E_Score2 (kcal/mol)	Compounds	E_Score2 (kcal/mol)
MOL01	−14.81	MOL01	−9.82	MOL01	−9.56
MOL06	−12.12	MOL02	−9.80	MOL03	−12.15
MOL07	−12.37	MOL03	−9.93	MOL06	−10.77
MOL16	−12.16	MOL05	−9.94	MOL07	−12.91
MOL23	−12.57	MOL06	−8.89	MOL08	−11.53
MOL32	−12.13	MOL07	−9.32	MOL26	−9.53
MOL33	−13.17	MOL13	−8.99	MOL27	−10.18
MOL34	−13.32	MOL30	−8.63	MOL30	−11.47
MOL36	−12.24	MOL33	−9.30	MOL33	−10.06
MOL49	−13.68	MOL37	−8.63	MOL49	−11.14

**Table 5:** HOMO and LUMO energy values (eV) of potential compounds.

Compound	HOMO	LUMO	HOMO-LUMO Gap
MOL17	−6.27	−2.14	4.14
MOL01	−6.44	−2.30	4.14
MOL02	−6.22	−2.09	4.13
MOL38	−5.96	−2.08	3.88
MOL08	−6.03	−1.90	4.13
MOL05	−6.17	−2.30	3.86
Gallic acid	−6.52	−1.59	4.93

**Table 6:** Bond dissociation enthalpies of –OH groups and ionization potentials of compounds.

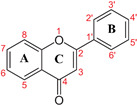				
Compound	BDE (kcal/mol)			IP (eV)
	4′-OH	5-OH	7-OH	
MOL01	81.12	97.36	87.03	170.52
MOL02	-	98.90	86.50	172.40
MOL05	-	92.00	-	172.36
MOL08	74.39	91.24	84.48	163.09
MOL17	81.97	98.86	86.70	174.80
MOL38	-	90.05	-	169.80
Gallic acid	92.63 (4-OH)			180.62

## Data Availability

The original contributions presented in this study are included in the article/Supplementary Materials. Further inquiries can be directed to the corresponding author.

## Supplementary Materials

The following supporting information can be downloaded at: https://www.mdpi.com/article/10.3390/molecules31040630/s1, Table S1: Inhibitory activity of *O. corniculata* extracts and fractions against α-glucosidase and α-amylase; Table S2: Antioxidant activity of *O. corniculata* extracts and fractions evaluated by DPPH and ABTS assays; Table S3: List of 130 compounds identified from *O. corniculata* L. based on literature review and screening results for drug-likeness and oral bioavailability; Table S4: List of gene IDs of 956 common targets; Table S5: List of 86 hub proteins in the protein–protein interaction (PPI) network; Table S6: Cartesian coordinates of MOL01 optimized using DFT calculations; Table S7: Cartesian coordinates of MOL02 optimized using DFT calculations; Table S8: Cartesian coordinates of MOL05 optimized using DFT calculations; Table S9: Cartesian coordinates of MOL08 optimized using DFT calculations; Table S10: Cartesian coordinates of MOL17 optimized using DFT calculations; Table S11: Cartesian coordinates of MOL38 optimized using DFT calculations; Table S12: Cartesian coordinates of gallic acid optimized using DFT calculations; Figure S1. KEGG pathway enrichment analysis of the identified targets; Figure S2. AGE–RAGE signaling pathway in diabetic complications identified by KEGG pathway analysis; Figure S3. Cardiomyopathy pathway in diabetic complications identified by KEGG pathway analysis; Figure S4. KEGG insulin resistance pathway; Figure S5. Chemical carcinogenesis—reactive oxygen species (ROS) pathway identified by KEGG analysis.
