# Supplementary material for: Beyond α-Glucosidase and α-Amylase Inhibition: Integrated In Vitro and Multi-Scale In Silico Insights into the Antidiabetic and Antioxidant Mechanisms of Oxalis corniculata L. Aerial Parts
Source: Molecules. 2026 Feb 12;31(4):630. doi: 10.3390/molecules31040630 (PMC12942679; doi:10.3390/molecules31040630)
Supplement: Supplementary file 1 [file molecules-31-00630-s001.zip › molecules-4117910-supplementary.pdf]

Beyond α-glucosidase and α-amylase inhibition: integrated *in vitro* and multi-scale *in silico* insights into the antidiabetic and antioxidant mechanisms of *Oxalis corniculata* L. aerial parts

Thi-Van-Anh Nguyen [1], Duong Quang Quy [1,2], Nguyen Thanh Tung [3], Nguyen Thu Huyen [1], Pham Le Minh [1], Nguyen T. Minh Huyen [1], Tue-Tam Ho [3], Nguyen Thi Thu Ha [4], Gerardo M. Casanola-Martin [2], Bakhtiyor Rasulev [2] and Hai Pham-The [1,*]

[1] University of Science and Technology of Hanoi (USTH), Vietnam Academy of Science and Technology, 18-Hoang Quoc Viet, Nghia Do, Hanoi 10000, Vietnam
[2] Department of Coatings and Polymeric Materials, North Dakota State University, Fargo, ND 58102, USA
[3] Hanoi University of Pharmacy, 13-15 Le Thanh Tong, Cua Nam, Hanoi 10000, Vietnam
[4] Institute of Chemistry (ICH), Vietnam Academy of Science and Technology, 18-Hoang Quoc Viet, Nghia Do, Hanoi 10000, Vietnam

* Correspondence: hpham.phd@gmail.com (H.P-T)

Supplementary materials:

Table S1. Inhibitory activity of *O. corniculata* extracts and fractions against α-glucosidase and α-amylase.

Sample	α-Glucosidase IC_{50} (mg/mL)	α-Amylase IC_{50} (mg/mL)
EtOH extract	0.135 ± 0.003	0.021 ± 0.003
n-Hex fraction	0.225 ± 0.003	0.062 ± 0.004
DCM fraction	0.260 ± 0.002	0.081 ± 0.002
EtOAc fraction	0.097 ± 0.004	0.015 ± 0.001
AqExt fraction	0.114 ± 0.002	0.032 ± 0.005
Acarbose	0.397 ± 0.003	0.013 ± 0.003

Data are presented as mean ± SD (n = 3).

Table S2. Antioxidant activity of *O. corniculata* extracts and fractions evaluated by DPPH and ABTS assays.

Sample	DPPH IC_{50} (mg/mL)	ABTS IC_{50} (mg/mL)
EtOH extract	0.0265 ± 0.003	0.0390 ± 0.004
n-Hex fraction	0.0659 ± 0.004	0.0656 ± 0.005
DCM fraction	0.0509 ± 0.005	0.0495 ± 0.005
EtOAc fraction	0.0249 ± 0.005	0.0202 ± 0.004
AqExt fraction	0.0357 ± 0.004	0.0330 ± 0.005
Ascorbic acid	0.0075 ± 0.003	
Trolox		0.0075 ± 0.003

Data are presented as mean ± SD (n = 3).

Table S3. List of 113 compounds identified from *Oxalis corniculata* L. based on literature review and screening results for drug-likeness and oral bioavailability

No.	Compound	TCMSP		SwissADME	
		OB (%)	DL	GI	"yes" DL
1	Stearic acid	17.83	0.14	High	1
2	*p*-Hydroxybenzoic acid	30.15	0.03	High	3
3	Vanillic acid	35.47	0.04	High	4
4	Syringic acid	47.78	0.06	High	4
5	Isoorientin	23.3	0.76	Low	0
6	Isovitexin	31.29	0.72	Low	2
7	Swertisin	5.75	0.75	Low	2
8	Acacetin	34.97	0.24	High	5
9	Apigenin 7,4'-dimethyl ether	27.12	0.27	High	5
10	Dillenetin	21.33	0.34	High	5
11	Corniculatin A	-	-	Low	0
12	Oxalic acid	29.68	0.01	High	3
13	Beta-sitosterol	36.91	0.75	Low	2
14	Betulin	15.48	0.78	Low	2
15	3,5,2'-Trihydroxy-7,5'-dimethoxyflavone	-	-	High	5
16	Apigenin	23.06	0.21	High	5
17	Gallic acid	31.69	0.04	High	3
18	Harmine	56.8	0.13	High	5
19	Harmaline	-	-	High	5
20	2-heptadecyl-5-methoxy-phenol	-	-	Low	1
21	Phlorizin	2.88	0.6	Low	2
22	Bilirubin	-	-	Low	0
23	Embelin	37.72	0.18	High	4
24	Quercitrin	4.04	0.74	Low	1
25	Parthenin	23.18	0.19	High	5
26	Petunidin	-	-	High	5
27	Octanal	19.07	0.01	High	4
28	Lecanoric acid	-	-	High	5
29	Sphinganine	-	-	High	3
30	Ophiobolin A	-	-	High	5
31	Phytosphingosine	-	-	High	3
32	*N*-acetylsphinganine	-	-	High	3
33	1-Linolenoyl lysolecithin	-	-	High	3
34	(+)-Larixol	-	-	High	3
35	Sebacic acid	16.23	0.05	High	5
36	Malvidin	-	-	High	5
37	Docosanedioic acid	16.2	0.36	Low	1
38	Apigetrin	9.68	0.74	Low	2
39	Rhapontin	-	-	Low	2
40	oleic acid	33.13	0.14	High	1
41	linoleic acid	41.9	0.14	High	1
42	linolenic acid	45.01	0.15	High	1
43	4'-O-Methylvitexin	-	-	Low	2
44	4'-OMeiso vitexin	-	-	Low	2
45	3',4'-diOMe orientin	-	-	Low	1
46	5-hydroxy-7,8-dimethoxyflavone	44.09	0.25	High	5

47	5-O-Desmethylnobiletin	-	-	High	5
48	5-hydroxy-3,6,7,4'-tetramethoxyflavone	8.2	0.4	High	5
49	4',5-hydroxy-3,6,7-trimethoxyflavone	24.96	0.36	High	5
50	3,3',5-trihydroxy-4'-methoxyflavone 7-O-β-D-glucopyranoside	-	-	Low	1
51	Tridecane	17.89	0.03	Low	4
52	Tetradecane	15.94	0.04	Low	2
53	Hexadecane	12.32	0.06	Low	1
54	Heptadecane	8.64	0.07	Low	1
55	Pentatriacontane	7.97	0.42	Low	1
56	1-Octene	39.25	0.01	Low	3
57	1-Dodecene	17.74	0.02	Low	4
58	4-Tetradecene	-	-	Low	4
59	1-Nonadecene	5.23	0.11	Low	1
60	1-Docosene	16.74	0.18	Low	1
61	Linalyl anthranilate	-	-	High	5
62	ethyl N-(heptan-4-ylideneamino)carbamate	-	-	High	5
63	Palmitic acid	19.3	0.1	High	3
64	1-Hexacosanol	10.95	0.35	Low	1
65	*E*-14-Hexadecenal	30.25	0.08	High	3
66	*E*-15-Heptadecenal	-	-	High	2
67	*p*-Coumaroyl hexose	9.8	0.26	Low	3
68	Sinapic acid	64.15	0.08	High	5
69	*p*-Coumaric acid	43.29	0.04	High	4
70	Caffeic acid	25.76	0.05	High	4
71	Kaempferol-3-(*p*-coumaroyldiglucoside)-7-glucoside	-	-	Low	0
72	Chlorogenic acid	11.93	0.33	Low	1
73	Kaempferol 3-glucuronide	-	-	Low	1
74	Vitexin 6''-O-malonyl 2''-O-xyloside	-	-	Low	0
75	Isorhamnetin-3-caffeoyl-7-glucoside	-	-	Low	0
76	Quercetin-3-(caffeoyldiglucoside)-7-glucoside	-	-	Low	0
77	Kaempferol 3-sophorotrioside	-	-	Low	0
78	Eschweilenol C	-	-	Low	1
79	Phytofluene	-	-	Low	1
80	Pheophorbide B	-	-	Low	1
81	Violaxanthin	17.68	0.51	Low	1
82	Pheophytin B	-	-	Low	1
83	Hydroxy-pheophytin B	-	-	Low	1
84	All-E-neoxanthin	28.01	0.51	Low	1
85	(10S)-Hydroxypheophytin A	-	-	Low	0
86	Hydroxy-pheophytin A'	-	-	Low	1
87	Pheophytin A	-	-	Low	0
88	All-E-lutein	22.59	0.55	Low	0
89	9-*cis*-Lutein	-	-	Low	0
90	Chlorophyll B	-	-	Low	1
91	13-Hydroxy-chlorophyll B	-	-	Low	0
92	Chlorophyl B'	-	-	Low	1
93	Chlorophyl A	-	-	Low	0
94	Eburicoic acid	38.7	0.81	Low	2
95	24-methylenecholest-4-ene-3β,6β-diol	-	-	High	2

96	Trametenolic acid	-	-	Low	2
97	Vitexin 2''-O-beta-D-glucoside	-	-	Low	0
98	Tartaric acid	-	-	Low	3
99	Citric acid	56.22	0.05	Low	2
100	Ascorbic acid	13.34	0.04	High	3
101	Maltose	1.8	0.24	Low	0
102	α-D-glucopyranoside, O-α-*D*-glucopyranosyl-(1-3)-α-D-fructofuranosyl	-	-	Low	0
103	Squalene	33.55	0.42	High	1
104	α-Tocopherol	14.26	0.55	Low	1
105	Limonene dioxide	-	-	High	4
106	Tectorigenin	28.41	0.27	High	5
107	Irigenin	1.95	0.41	High	5
108	Iristectorigenin A	63.36	0.34	High	5
109	Lespedin	7.97	0.79	Low	0
110	Rotenone	-	-	High	5
111	2-Pentylfuran	54.59	0.02	High	3
112	Pyruvic acid	54.46	0.01	High	3
113	Malic acid	-	-	High	3

"-" : No data available.

Table S4. List of gene IDs of 956 common targets

ABCB1	ABCB11	ABCC1	ABCC4	ABCC9	ABCG2	ACACA	ACACB
ACAT1	ACE	ACE2	ACLY	ACP1	ACR	ACVR2B	ACHE
ADA	ADAM17	ADH1B	ADH1C	ADORA1	ADORA2B	ADORA3	ADRA1A
ADRA1B	ADRA1D	ADRA2A	ADRA2B	ADRA2C	ADRB1	ADRB2	ADRB3
AGPAT2	AGTR1	AGXT2	AHR	AHSA1	AKR1A1	AKR1B1	AKR1B10
AKR1C2	AKT1	AKT2	ALB	ALDH1A2	ALDH2	ALK	ALOX12
ALOX15	ALOX5	ALOX5AP	ALPL	AMPD1	AMY1A	ANPEP	AOC3
APC	APOB	APP	AR	ARG1	ATIC	ATP12A	ATP2A1
ATP4A	AVPR2	BACE1	BACE2	BAD	BAX	BCAT1	BCL2
BCL2A1	BCL2L1	BCHE	BDKRB2	BHMT	BIRC2	BMP4	BRAF
BRD2	BRD4	BRD9	BRS3	CA2	CA4	CA5A	CACNA1C
CACNA1D	CALM1	CAMK1D	CAMK2A	CAMK2G	CAMKK2	CAPN1	CAPN2
CASP1	CASP3	CASP7	CASP8	CASP9	CASR	CAT	CBR1
CBS	CCKAR	CCKBR	CCL16	CCND1	CCND2	CCND3	CCR1
CCR2	CCR5	CD38	CD4	CD40LG	CD81	CDC42	CDK11A
CDK2	CDK4	CDK5	CDK6	CDK9	CDKN1A	CDKN1B	CDKN1C
CDKN2A	CEL	CELA1	CES1	CETP	CFD	CFLAR	CFTR
CISD1	CLK2	CNR1	CNR2	COL1A1	COMT	CPA1	CPT1A
CPT1B	CPT2	CREB1	CREBBP	CRHR1	CRHR2	CSNK2A1	CTNNB1
CTSB	CTSC	CTSD	CTSH	CTSL	CTSS	CTRB1	CXCL12
CXCR1	CXCR3	CXCR4	CYCS	CYP11B2	CYP17A1	CYP19A1	CYP1A1
CYP1A2	CYP1B1	CYP24A1	CYP27B1	CYP2A6	CYP2C19	CYP2C8	CYP2C9
CYP2D6	CYP3A4	CHEK2	CHRM3	CHRNA4	CHRNB4	CHUK	DAPK2
DBH	DGAT1	DHCR7	DMPK	DNTT	DPP4	DPP9	DRD1
DRD2	DRD3	DRD4	DRD5	DYRK1A	DYRK1B	DYRK3	EBP
ECE1	EDNRA	EDNRB	EGFR	EIF2AK2	EIF2AK3	EIF2AK4	ELANE

ELAVL1	ENGASE	EP300	EPAS1	EPHA4	EPHB1	EPHB2	EPHX1
EPHX2	ERBB2	ERG	ERN1	ESR1	ESR2	ESRRA	ESRRB
ESRRG	F12	F2	F2R	F2RL3	F3	F7	F9
FAAH	FABP1	FABP2	FABP3	FABP4	FABP5	FADS1	FAP
FASLG	FASN	FBP1	FEN1	FFAR1	FFAR4	FGF1	FGF2
FGFR1	FGFR2	FLT1	FOLH1	FOS	FTO	G6PC	G6PC1
G6PD	GAA	GABRA2	GABRA4	GABRG2	GANAB	GANC	GAPDH
GC	GCG	GCGR	GCK	GFAP	GFER	GHSR	GLA
GLB1	GLI2	GLO1	GLS	GLUL	GNAO1	GOT1	GOT2
GPBAR1	GPR119	GPR35	GPR39	GPR55	GPT	GRK2	GSK3A
GSK3B	GSR	GSTA1	GSTK1	GSTM1	GSTP1	GUSB	GYS1
GIP	HCAR2	HDAC3	HDAC4	HDAC7	HDAC9	HGF	HIF1A
HLA-A	HMGB1	HMGCR	HMOX1	HNF4A	HPGDS	HPSE	HRAS
HSD11B1	HSD11B2	HSD17B3	HSP90AA1	HSP90B1	HSPA1A	HSPA8	HSPD1
HTR1A	HTR1B	HTR1D	HTR2A	HTR2B	HTR2C	HTR3B	HTR4
HTR7	IARS1	ICAM1	IFNG	IGF1R	IGF2	IGF2R	IGFBP1
IGFBP2	IGFBP3	IGFBP4	IGFBP5	IKBKB	IKBKE	IL10	IL13
IL1B	IL2	IL4	IL6	ILK	INS	INSR	IRAK4
ITGA2B	ITGB2	ITGB3	ITGB6	JAK1	JAK2	JUN	KCNB1
KCND3	KCNJ1	KCNJ11	KCNJ3	KCNJ5	KCNJ6	KCNK3	KCNMA1
KCNQ1	KDM1A	KDR	KIF11	KISS1R	KLF5	KLK1	KLK5
LDHA	LDLR	LIMK1	LIPA	LIPE	LIPG	LNPEP	LOXL2
LPAR1	LPAR2	LPAR3	LRP6	LTF	LY96	LYPLA1	LYZ
MAK	MALT1	MAOA	MAOB	MAP2K1	MAP2K7	MAP3K1	MAP3K8
MAP4K5	MAPK1	MAPK10	MAPK14	MAPK3	MAPK7	MAPK8	MAPK9
MAPT	MAX	MB	MC4R	MC5R	MDH2	MDM2	ME1
MET	METAP2	MGAM	MGAT2	MGLL	MIF	MME	MMP1
MMP12	MMP13	MMP14	MMP2	MMP26	MMP3	MMP7	MMP8
MMP9	MPI	MPO	MRGPRX1	MS4A2	MT-ND1	MT-ND2	MT-ND3
MT-ND4	MT-ND5	MTNR1A	MTNR1B	MTOR	MTTP	MTR	MYLK
NAMPT	NCOA1	NDUFA12	NDUFB6	NDUFS4	NEK2	NFE2L2	NFKB1
NFKBIA	NISCH	NLRP3	NNMT	NOD1	NOD2	NOS1	NOS2
NOS3	NOX4	NPC1	NPC1L1	NPEPPS	NPY1R	NPY2R	NPY5R
NQO1	NR0B2	NR1H2	NR1H3	NR1H4	NR1I2	NR1I3	NR3C1
NR3C2	NR4A1	NR4A2	NSD2	NTRK1	NTRK2	NGFR	OGA
OPRM1	OXCT1	OXER1	P2RX3	P2RX7	P2RY1	P2RY12	P4HB
PADI1	PADI4	PAK1	PAM	PARP1	PASK	PCSK7	PCYT1A
PDE2A	PDE3A	PDE3B	PDE4B	PDE4D	PDE5A	PDGFRB	PDHB
PDK4	PEPD	PER2	PFKFB3	PGC	PGF	PGR	PIK3C2G
PIK3CA	PIK3CB	PIK3CD	PIK3CG	PIK3R1	PIM3	PIN1	PKLR
PKM	PKN2	PLA2G10	PLA2G1B	PLA2G2A	PLA2G4A	PLA2G6	PLA2G7
PLAT	PLAUR	PLCG1	PLD1	PLG	PLK1	PON1	PPARA
PPARD	PPARG	PPIA	PPP2CA	PREP	PRKAA2	PRKAB1	PRKCA
PRKCB	PRKCD	PRKCE	PRKCI	PRKCQ	PRKCZ	PRKD1	PRMT1
PSEN1	PSMD3	PTAFR	PTEN	PTER	PTGDR2	PTGER3	PTGES2
PTGS1	PTGS2	PTK2B	PTPN1	PTPN11	PTPN2	PTPN22	PTPN5
PTPN6	PTPRB	PTPRC	PTPRF	PTPRS	PYGM	RAC1	RAF1
RAMP1	RARB	RBP4	RELA	REN	RHO	RHOA	RIPK2
ROCK1	RORA	RORB	RORC	RPS6KA2	RPS6KA3	RPS6KB1	RXRA
RXRB	RXRG	SCD	SCN2A	SCNN1A	SDHA	SELE	SELL
SELP	SERPINA6	SERPINE1	SHBG	SHH	SI	SIGMAR1	SIRT1

SIRT2	SIRT3	SIRT5	SLC10A2	SLC16A1	SLC16A7	SLC18A2	SLC22A1
SLC22A12	SLC22A2	SLC22A3	SLC22A6	SLC22A8	SLC25A20	SLC2A2	SLC2A3
SLC2A4	SLC47A1	SLC5A1	SLC5A2	SLC5A4	SLC5A5	SLC6A2	SLC6A4
SLC8A1	SLC9A1	SMAD3	SNCA	SOAT1	SPHK1	SPHK2	SRC
SRD5A1	SRD5A2	SREBF2	SSTR2	SSTR4	ST3GAL3	ST6GAL1	STAT1
STAT3	STK33	STK4	STS	SUCLA2	SYK	TAAR1	TACR3
TAS1R3	TCF4	TEK	TEP1	TERT	TF	TGFB1	TGFBR1
TGFBR2	TGM2	TIMP3	TKFC	TKT	TLR1	TLR2	TLR4
TLR9	TNF	TNFRSF1A	TNKS2	TNNT2	TOP1	TOP2A	TP53
TPO	TSPO	TTR	TYK2	TYMP	TH	TRPA1	TRPM2
TRPM5	TRPV1	UGCG	UGT2B7	UTS2R	VCAM1	VDR	VEGFA
XDH	YWHAG	ABL1	ADAM10	ADH5	ADORA2A	AHCY	AKR1C1
AKR1C3	ALDH1A1	ALDH3A1	ALDH5A1	ALDH9A1	ALKBH3	AMPD3	APEX1
ASAH1	ATF1	ATG4B	ATR	AURKB	BCL2L2	BRPF1	BTK
C3AR1	C5AR1	CA3	CAMK2D	CASP2	CASP6	CCNA2	CCNB1
CCR3	CDC25A	CDC25C	CDK1	CES2	CRYAB	CTSG	CTSV
CTH	CXCR2	CYP26A1	CHAT	CHEK1	CHKA	CHRNA7	CHRNB2
DAO	DAPK1	DDO	DHCR24	DHFR	DHODH	DNMT3B	DUSP3
EEF1A1	EEF1A2	EGLN1	EGLN3	EIF6	EPHA3	ERCC1	ERCC5
EZH2	F10	FLT3	FYN	GATM	GLUD1	GLUD2	GNAI1
GNB1	GNG2	GRIN1	GRIN2A	GRIN2B	GRM5	GSTM2	GSTO1
HAO1	HCK	HCRTR1	HDAC1	HDAC10	HDAC11	HDAC2	HDAC5
HDAC6	HDAC8	HIF1AN	HRH2	HRH4	HSD17B2	HSF1	HSP90AB1
HTT	ICMT	IDH1	IDH2	IDO1	IKBKG	ITGA4	ITGAL
ITGAV	ITGB1	ITGB5	KCNA3	KCNA5	KCNE1	KCNK9	KCNH2
KCNH3	KIT	KMO	LCK	LDHB	LIG1	LRRK2	LTB4R
LYN	MAP2	MAP3K14	MAP3K7	MAPK15	MAPKAPK2	MCL1	MDH1
MDM4	ME2	ME3	MECP2	MGMT	MGST1	MITF	MPEG1
MPG	MT-ND4L	MT-ND6	NDUFA1	NDUFA10	NDUFA11	NDUFA13	NDUFA2
NDUFA3	NDUFA4	NDUFA4L2	NDUFA5	NDUFA6	NDUFA7	NDUFA8	NDUFA9
NDUFAB1	NDUFAF1	NDUFAF2	NDUFAF3	NDUFAF4	NDUFB1	NDUFB10	NDUFB11
NDUFB2	NDUFB3	NDUFB4	NDUFB5	NDUFB7	NDUFB8	NDUFB9	NDUFC1
NDUFC2	NDUFS1	NDUFS2	NDUFS3	NDUFS5	NDUFS6	NDUFS7	NDUFS8
NDUFV1	NDUFV2	NDUFV3	NFS1	NLRP1	NQO2	NTSR1	OAT
ODC1	OPRK1	PABPC1	PARP2	PDE4A	PDGFRA	PDK1	PDK3
PGD	PGK1	PIM1	PIM2	PLAU	PLCG2	POLB	POLH
POLI	POLL	PRKACA	PRKAG1	PRKCG	PRKDC	PSEN2	PSMB5
PTGES	PTK2	PTPN12	QDPR	RAD51	RARA	RB1	RIPK1
RPA1	RPS6KA1	RPS6KB2	RUNX1T1	SCN5A	SHMT2	SLC16A4	SLC1A1
SLC37A4	SLC6A12	SLC6A3	SLC7A11	SMPD2	SOAT2	SQLE	ST14
STK3	TACR1	TBXA2R	TDP1	TLR8	TNNI3	TOP2B	TYR
TYRP1	TRPM8	TRPV4	UBE2D3	UQCRB	USP10	VAV1	VCP
WEE1	XIAP	XPO1	YARS1				

Table S5. List of 86 hub proteins in the protein–protein interaction (PPI) network.

No.	Gene ID	DC	EC	LAC	BC	CC	NC
1	SRC	120	0.173	30.617	2780.278	0.670	84.263
2	AKT1	114	0.169	29.719	2012.759	0.660	74.475
3	TP53	113	0.147	26.549	2986.067	0.658	77.059
4	MAPK3	110	0.162	28.527	1763.745	0.653	70.305
5	STAT3	104	0.163	31.250	1287.163	0.642	69.128
6	HSP90AA1	103	0.143	24.388	1926.044	0.640	59.426
7	EGFR	101	0.156	29.307	1416.507	0.635	64.062
8	MAPK1	97	0.141	24.887	1312.481	0.630	55.596
9	HRAS	92	0.142	27.783	1243.605	0.618	57.230
10	JUN	92	0.133	26.239	1463.556	0.622	55.718
11	CTNNB1	91	0.135	24.593	1256.456	0.618	50.679
12	PIK3R1	83	0.131	27.229	589.721	0.601	50.112
13	TNF	77	0.101	22.000	1180.272	0.592	45.675
14	PIK3CA	77	0.127	27.792	418.421	0.595	46.791
15	EP300	77	0.098	21.299	1268.206	0.598	45.526
16	RELA	74	0.114	24.108	792.664	0.593	40.193
17	VEGFA	71	0.120	26.930	468.048	0.589	41.678
18	MAPK8	69	0.109	22.000	611.026	0.583	34.265
19	IL6	68	0.099	22.471	754.430	0.577	39.513
20	CASP3	68	0.101	20.353	634.240	0.580	32.916
21	ESR1	67	0.111	24.836	589.687	0.582	36.866
22	RHOA	65	0.105	22.708	444.180	0.566	33.032
23	CREBBP	65	0.083	19.538	820.813	0.580	36.621
24	JAK2	63	0.100	20.952	474.324	0.569	30.276
25	FYN	63	0.102	24.730	340.619	0.557	36.399
26	PTPN11	62	0.105	24.903	268.730	0.560	34.429
27	GAPDH	62	0.086	18.742	1114.016	0.573	30.373
28	MAPK14	62	0.105	21.774	457.923	0.572	29.846
29	INS	61	0.095	19.705	775.899	0.575	29.010
30	PLCG1	61	0.093	21.377	341.432	0.552	31.648
31	PTK2	60	0.106	26.967	197.600	0.560	35.529
32	LYN	60	0.102	23.567	338.622	0.562	32.502
33	LCK	60	0.103	26.500	190.210	0.556	35.838
34	PTEN	58	0.102	23.172	367.228	0.565	29.710
35	RAC1	58	0.096	23.172	283.446	0.548	31.495
36	HIF1A	57	0.091	21.895	496.906	0.569	30.313
37	IL1B	56	0.082	20.964	341.878	0.558	31.267
38	STAT1	56	0.099	22.429	274.301	0.566	27.492
39	TLR4	55	0.084	19.891	363.407	0.557	28.963
40	PRKCA	54	0.079	16.037	443.093	0.554	23.230
41	CDC42	54	0.093	22.556	186.482	0.549	28.649
42	NFKB1	53	0.089	20.491	227.738	0.558	25.637
43	JAK1	51	0.084	18.941	249.231	0.541	24.251
44	ERBB2	51	0.088	19.333	405.580	0.556	23.172
45	CCND1	51	0.084	21.843	230.361	0.556	28.092
46	NFKBIA	50	0.084	20.080	256.667	0.557	24.646
47	AR	50	0.080	18.400	340.415	0.553	23.508
48	CXCL12	49	0.082	20.776	199.709	0.543	26.075

49	CREB1	47	0.073	15.872	296.574	0.550	20.159
50	MTOR	47	0.083	17.957	238.394	0.548	20.577
51	HDAC1	47	0.061	17.362	330.533	0.539	23.869
52	FOS	46	0.082	20.304	170.712	0.553	23.535
53	PPARG	46	0.070	17.130	351.957	0.553	22.101
54	IL2	46	0.084	21.087	129.354	0.540	24.100
55	SIRT1	46	0.064	16.957	252.732	0.544	23.116
56	MDM2	46	0.065	16.000	320.059	0.547	20.569
57	SYK	45	0.071	17.333	195.669	0.525	21.254
58	GNB1	45	0.070	18.222	197.957	0.529	23.155
59	AKT2	44	0.075	16.636	170.189	0.544	19.046
60	HSP90AB1	44	0.066	14.682	237.755	0.540	19.155
61	PRKCZ	43	0.079	19.953	101.823	0.541	22.181
62	ALB	43	0.063	15.907	385.094	0.544	20.794
63	RAF1	43	0.075	16.930	134.798	0.533	19.019
64	PRKCD	43	0.075	17.767	154.294	0.535	20.323
65	CXCR4	42	0.069	17.952	238.592	0.529	20.944
66	CDKN1A	42	0.065	17.714	168.676	0.543	21.724
67	BCL2L1	41	0.070	16.439	149.983	0.544	19.440
68	NR3C1	41	0.070	17.220	128.060	0.545	19.724
69	MAP2K1	40	0.069	15.350	144.834	0.539	17.305
70	SMAD3	40	0.058	14.000	221.471	0.535	17.007
71	FGF2	40	0.066	17.550	114.204	0.533	20.449
72	CD4	40	0.059	16.100	191.018	0.519	19.123
73	ITGB1	39	0.066	17.077	100.371	0.519	18.877
74	MMP9	39	0.064	17.590	185.780	0.538	21.232
75	TLR2	39	0.056	15.077	147.910	0.525	18.419
76	PDGFRB	39	0.070	18.564	100.730	0.518	20.484
77	ABL1	39	0.060	13.846	212.860	0.535	16.342
78	RPS6KB1	38	0.066	14.947	116.395	0.538	16.167
79	GNAI1	38	0.067	18.526	99.782	0.526	21.179
80	CDK2	38	0.055	16.579	159.312	0.535	20.126
81	IKBKB	37	0.061	15.514	98.554	0.537	17.491
82	RB1	37	0.058	16.919	108.502	0.529	19.417
83	TGFB1	36	0.058	14.000	142.951	0.532	16.400
84	CASP8	35	0.059	14.629	99.100	0.534	16.436
85	ITGAV	34	0.055	14.000	110.426	0.516	15.820
86	PPP2CA	34	0.059	14.588	104.873	0.527	16.036

Table S6. Cartesian coordinates of MOL01 optimized using DFT calculations.

No.	Atom	Coordinates (Å)		
		X	Y	Z
1	O1	3.4739	-0.7052	-0.8454
2	O2	3.0040	1.8624	1.7818
3	O3	5.6097	0.8850	2.3169
4	O4	6.9716	-0.1605	0.0481
5	O5	5.0936	-3.0219	-1.1382
6	O6	0.9008	2.3969	-0.9352
7	O7	1.6450	-2.1106	0.4952
8	O8	-2.7587	-0.5212	0.1093
9	O9	-1.5395	3.1686	-1.1601
10	O10	-8.9997	-1.3249	0.5168
11	C11	3.4627	0.6628	1.1659
12	C12	4.9771	0.8081	1.0419
13	C13	2.8389	0.4443	-0.2359
14	C14	5.5900	-0.3805	0.3061
15	C15	4.8928	-0.5690	-1.0459
16	C16	1.3499	0.1871	-0.1889
17	C17	5.3643	-1.7992	-1.8172
18	C18	0.4435	1.2024	-0.5278
19	C19	0.8259	-1.0701	0.2059
20	C20	-0.9602	0.9896	-0.4305
21	C21	-0.5489	-1.3029	0.3175
22	C22	-1.4154	-0.2662	0.0016
23	C23	-1.9126	2.0341	-0.7681
24	C24	-3.6817	0.4366	-0.1965
25	C25	-3.3049	1.6792	-0.6111
26	C26	-5.0601	-0.0393	-0.0105
27	C27	-6.1510	0.6514	-0.5749
28	C28	-5.3251	-1.1989	0.7389
29	C29	-7.4544	0.2124	-0.3895
30	C30	-6.6295	-1.6460	0.9307
31	C31	-7.6998	-0.9403	0.3689
32	H32	3.2375	-0.2033	1.8050
33	H33	5.1889	1.7236	0.4669
34	H34	3.0302	1.3333	-0.8481
35	H35	5.4531	-1.2902	0.9079
36	H36	5.0822	0.3191	-1.6691
37	H37	6.4490	-1.7356	-1.9318
38	H38	4.9049	-1.7932	-2.8141
39	H39	2.0494	1.8089	1.9259
40	H40	5.2113	1.6222	2.8012
41	H41	7.3894	0.0748	0.8891
42	H42	-0.9153	-2.2770	0.6164
43	H43	4.1678	-3.2563	-1.2795
44	H44	0.0928	2.9631	-1.1235
45	H45	2.5351	-1.9045	0.1417

46	H46	-4.0445	2.4411	-0.8184
47	H47	-5.9817	1.5330	-1.1836
48	H48	-4.5040	-1.7512	1.1804
49	H49	-8.2918	0.7415	-0.8313
50	H50	-6.8139	-2.5418	1.5191
51	H51	-9.0552	-2.1302	1.0487

Table S7. Cartesian coordinates of MOL02 optimized using DFT calculations.

No.	Atom	Coordinates (Å)		
		X	Y	Z
1	O1	-0.3736	0.5978	-0.0656
2	O2	-4.6307	-1.6206	0.1129
3	O3	-2.4032	-2.9450	0.1536
4	O4	-4.3262	3.1422	-0.1321
5	O5	5.9203	0.0806	-0.0791
6	C6	-2.4751	-0.5802	0.0270
7	C7	-1.7457	0.6195	-0.0428
8	C8	0.3113	-0.5811	-0.0071
9	C9	1.7676	-0.3812	-0.0320
10	C10	-1.7810	-1.8558	0.0870
11	C11	-0.3371	-1.7771	0.0761
12	C12	-3.8956	-0.5023	0.0443
13	C13	-2.3540	1.8672	-0.0966
14	C14	-3.7526	1.9059	-0.0783
15	C15	-4.5275	0.7404	-0.0089
16	C16	2.6450	-1.4495	-0.3140
17	C17	2.3263	0.8795	0.2281
18	C18	4.0176	-1.2649	-0.3210
19	C19	3.7076	1.0776	0.2260
20	C20	4.5630	0.0020	-0.0480
21	C21	6.5423	1.3364	0.1814
22	H22	0.2181	-2.7023	0.1551
23	H23	-1.7725	2.7785	-0.1540
24	H24	-5.6125	0.7816	0.0050
25	H25	2.2504	-2.4321	-0.5490
26	H26	1.6745	1.7181	0.4435
27	H27	4.6918	-2.0846	-0.5453
28	H28	4.0992	2.0649	0.4387
29	H29	-3.9857	-2.3847	0.1440
30	H30	-5.2893	3.0621	-0.1165
31	H31	6.3065	1.6942	1.1907
32	H32	6.2413	2.0892	-0.5570
33	H33	7.6145	1.1567	0.1009

Table S8. Cartesian coordinates of MOL05 optimized using DFT calculations.

No.	Atom	Coordinates (Å)		
		X	Y	Z
1	O1	0.5855	-0.3027	0.0127
2	O2	-1.2767	-2.2802	0.0411
3	O3	-4.0127	-1.5650	0.1244
4	O4	-2.8809	3.0388	0.0373
5	O5	-0.3556	3.6741	-0.0401
6	C6	-1.1042	1.4247	0.0113
7	C7	-0.7334	0.0677	0.0208
8	C8	-1.6771	-0.9643	0.0556
9	C9	1.5713	0.6351	-0.0384
10	C10	-2.4861	1.7553	0.0411
11	C11	-0.0729	2.4530	-0.0302
12	C12	-3.0390	-0.6031	0.0593
13	C13	1.2903	1.9666	-0.0700
14	C14	-3.4394	0.7369	0.0632
15	C15	2.9151	0.0274	-0.0709
16	C16	4.0538	0.7808	0.2670
17	C17	3.0758	-1.3182	-0.4474
18	C18	5.3221	0.2062	0.2144
19	C19	4.3480	-1.8878	-0.4993
20	C20	5.4746	-1.1294	-0.1714
21	C21	-1.2092	-2.8951	1.3393
22	C22	-4.1446	-2.4101	-1.0332
23	H23	2.0847	2.6975	-0.1447
24	H24	-4.4956	0.9796	0.0785
25	H25	3.9493	1.8108	0.5911
26	H26	2.2036	-1.9080	-0.7056
27	H27	6.1910	0.7991	0.4833
28	H28	4.4582	-2.9257	-0.7991
29	H29	-2.0547	3.5947	0.0092
30	H30	6.4639	-1.5755	-0.2111
31	H31	-2.1884	-2.8755	1.8296
32	H32	-0.4672	-2.3899	1.9684
33	H33	-0.9012	-3.9286	1.1700
34	H34	-4.4306	-1.8108	-1.9057
35	H35	-4.9443	-3.1135	-0.7966
36	H36	-3.2158	-2.9497	-1.2362

Table S9. Cartesian coordinates of MOL08 optimized using DFT calculations.

No.	Atom	Coordinates (Å)		
		X	Y	Z
1	O1	1.1812	-2.4732	-0.3481
2	O2	5.0937	1.4176	-0.0783
3	O3	2.3569	2.1654	0.1548
4	O4	-0.0840	1.3842	0.1903
5	O5	5.7165	-1.1469	-0.4086
6	O6	-4.7699	1.4582	-1.1512
7	O7	-6.2645	-0.2761	0.4028
8	C8	1.7023	-0.1402	-0.0717
9	C9	-0.6452	-0.9304	-0.0391
10	C10	2.1151	-1.4729	-0.2620
11	C11	0.2932	0.1975	0.0361
12	C12	-2.1088	-0.7458	0.1131
13	C13	2.7017	0.8768	0.0002
14	C14	4.0481	0.5194	-0.0872
15	C15	3.4490	-1.8418	-0.3794
16	C16	-0.1311	-2.1719	-0.2436
17	C17	4.4063	-0.8307	-0.2913
18	C18	-2.7805	0.3041	-0.5371
19	C19	-2.8625	-1.6283	0.9011
20	C20	-4.1600	0.4604	-0.4311
21	C21	-4.2464	-1.4833	1.0108
22	C22	-4.9070	-0.4510	0.3424
23	C23	5.2190	2.2551	1.0892
24	C24	-5.3601	2.5174	-0.3836
25	H25	3.7389	-2.8730	-0.5369
26	H26	-0.7399	-3.0614	-0.3534
27	H27	-2.2367	1.0187	-1.1425
28	H28	-2.3686	-2.4218	1.4539
29	H29	-4.8174	-2.1761	1.6256
30	H30	1.3559	2.1803	0.2012
31	H31	6.2143	-0.3111	-0.3644
32	H32	4.3690	2.9352	1.1665
33	H33	5.2918	1.6392	1.9933
34	H34	6.1426	2.8191	0.9498
35	H35	-6.6585	-0.9734	0.9432
36	H36	-5.7626	3.2245	-1.1111
37	H37	-6.1676	2.1440	0.2531
38	H38	-4.5985	3.0159	0.2288

Table S10. Cartesian coordinates of MOL17 optimized using DFT calculations.

No.	Atom	Coordinates (Å)		
		X	Y	Z
1	O1	-0.0391	-0.6850	0.0531
2	O2	-4.1368	1.8191	-0.0864
3	O3	-1.8239	2.9896	-0.1375
4	O4	-4.1548	-2.9545	0.1358
5	O5	6.2426	-0.7548	-0.0317
6	C6	-2.0564	0.6343	-0.0199
7	C7	-1.4101	-0.6129	0.0395
8	C8	0.7226	0.4449	-0.0059
9	C9	2.1628	0.1467	0.0066
10	C10	-1.2779	1.8600	-0.0797
11	C11	0.1577	1.6827	-0.0810
12	C12	-3.4791	0.6531	-0.0276
13	C13	-2.1013	-1.8164	0.0923
14	C14	-3.4995	-1.7599	0.0834
15	C15	-4.1936	-0.5439	0.0245
16	C16	3.1049	1.1401	0.3255
17	C17	2.6360	-1.1428	-0.3055
18	C18	4.4684	0.8649	0.3171
19	C19	3.9963	-1.4265	-0.3167
20	C20	4.9197	-0.4213	-0.0069
21	H21	0.7748	2.5675	-0.1647
22	H22	-1.5825	-2.7653	0.1419
23	H23	-5.2790	-0.5109	0.0178
24	H24	2.7764	2.1360	0.6025
25	H25	1.9279	-1.9260	-0.5496
26	H26	5.1803	1.6459	0.5740
27	H27	4.3594	-2.4183	-0.5645
28	H28	-3.4417	2.5375	-0.1191
29	H29	-5.1106	-2.8103	0.1267
30	H30	6.7876	0.0111	0.1937

Table S11. Cartesian coordinates of MOL38 optimized using DFT calculations.

No.	Atom	Coordinates (Å)		
		X	Y	Z
1	O1	-0.2415	-0.7681	-0.0701
2	O2	5.2679	-0.2185	-0.0299
3	O3	4.0420	-2.7376	-0.1221
4	O4	3.6540	2.0421	0.0161
5	O5	1.2631	3.0235	0.0134
6	O6	-6.4134	-1.6507	-0.0070
7	C7	1.6694	0.6896	-0.0166
8	C8	1.1147	-0.5985	-0.0462
9	C9	-1.1050	0.2897	-0.0240
10	C10	-2.5053	-0.1594	-0.0300
11	C11	3.0854	0.8246	-0.0103
12	C12	0.8022	1.8539	0.0031
13	C13	1.9040	-1.7429	-0.0548
14	C14	-0.6291	1.5803	0.0233
15	C15	3.8991	-0.3176	-0.0483
16	C16	3.2942	-1.5958	-0.0511
17	C17	-2.8182	-1.4663	0.4084
18	C18	-3.5593	0.6523	-0.4835
19	C19	-4.1236	-1.9300	0.4088
20	C20	-5.1670	-1.1048	-0.0433
21	C21	-4.8760	0.1896	-0.4943
22	C22	5.8683	0.3410	-1.2110
23	C23	4.9419	-3.0013	0.9693
24	C24	-7.5172	-0.8729	-0.4618
25	H25	1.4632	-2.7319	-0.0753
26	H26	-2.0250	-2.1177	0.7561
27	H27	-3.3491	1.6522	-0.8382
28	H28	-5.6579	0.8438	-0.8604
29	H29	2.9035	2.7014	0.0194
30	H30	6.9459	0.3061	-1.0414
31	H31	5.5496	1.3762	-1.3577
32	H32	5.6146	-0.2635	-2.0899
33	H33	4.3762	-3.1141	1.9022
34	H34	5.6839	-2.2070	1.0716
35	H35	5.4300	-3.9457	0.7239
36	H36	-7.4042	-0.6025	-1.5185
37	H37	-7.6384	0.0359	0.1400
38	H38	-8.3953	-1.5078	-0.3413
39	H39	-4.3624	-2.9304	0.7544
40	O40	-1.5112	2.6258	0.0122
41	C41	-1.4732	3.5019	1.1563
42	H42	-0.5042	3.9987	1.2299
43	H43	-2.2617	4.2373	0.9882
44	H44	-1.6873	2.9368	2.0715

Table S12. Cartesian coordinates of gallic acid optimized using DFT calculations.

No.	Atom	Coordinates (Å)		
		X	Y	Z
1	O1	-3.0829	-0.1217	0.0001
2	O2	-1.8699	2.2959	0.0000
3	O3	-1.6446	-2.4314	0.0000
4	O4	3.1602	-1.0855	0.0000
5	O5	3.1764	1.1660	-0.0001
6	C6	1.0632	0.0316	0.0000
7	C7	-1.7178	-0.0361	0.0000
8	C8	-1.0464	1.1919	0.0000
9	C9	-0.9926	-1.2340	0.0000
10	C10	0.3411	1.2362	0.0000
11	C11	0.4007	-1.2034	0.0000
12	C12	2.5427	0.1249	-0.0001
13	H13	0.8794	2.1788	0.0000
14	H14	0.9521	-2.1350	-0.0001
15	H15	-3.4575	0.7723	0.0001
16	H16	-1.3487	3.1089	0.0001
17	H17	-2.6004	-2.2702	0.0000
18	H18	4.1154	-0.9085	0.0001

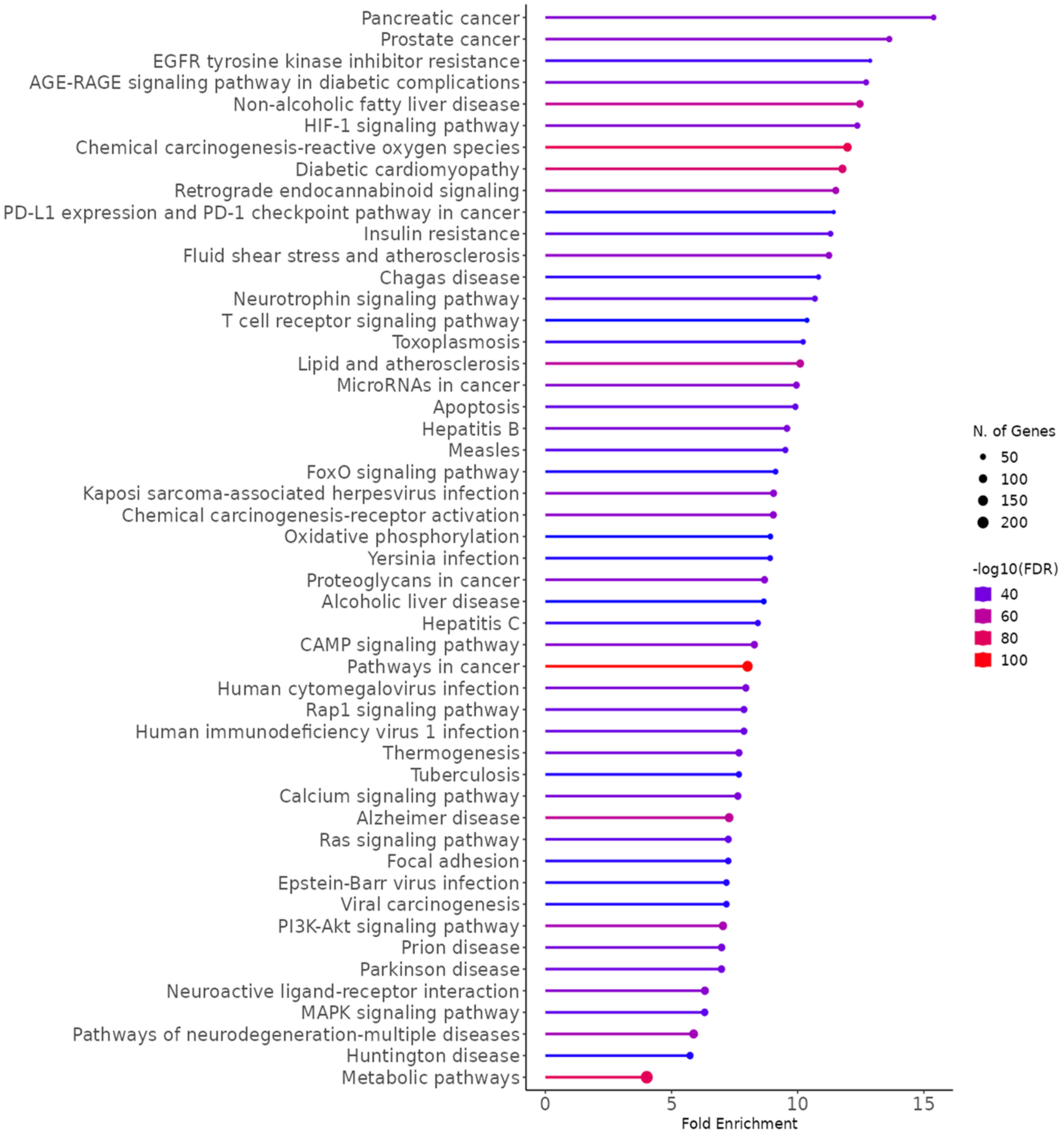

Figure S1. KEGG pathway enrichment analysis of the identified targets

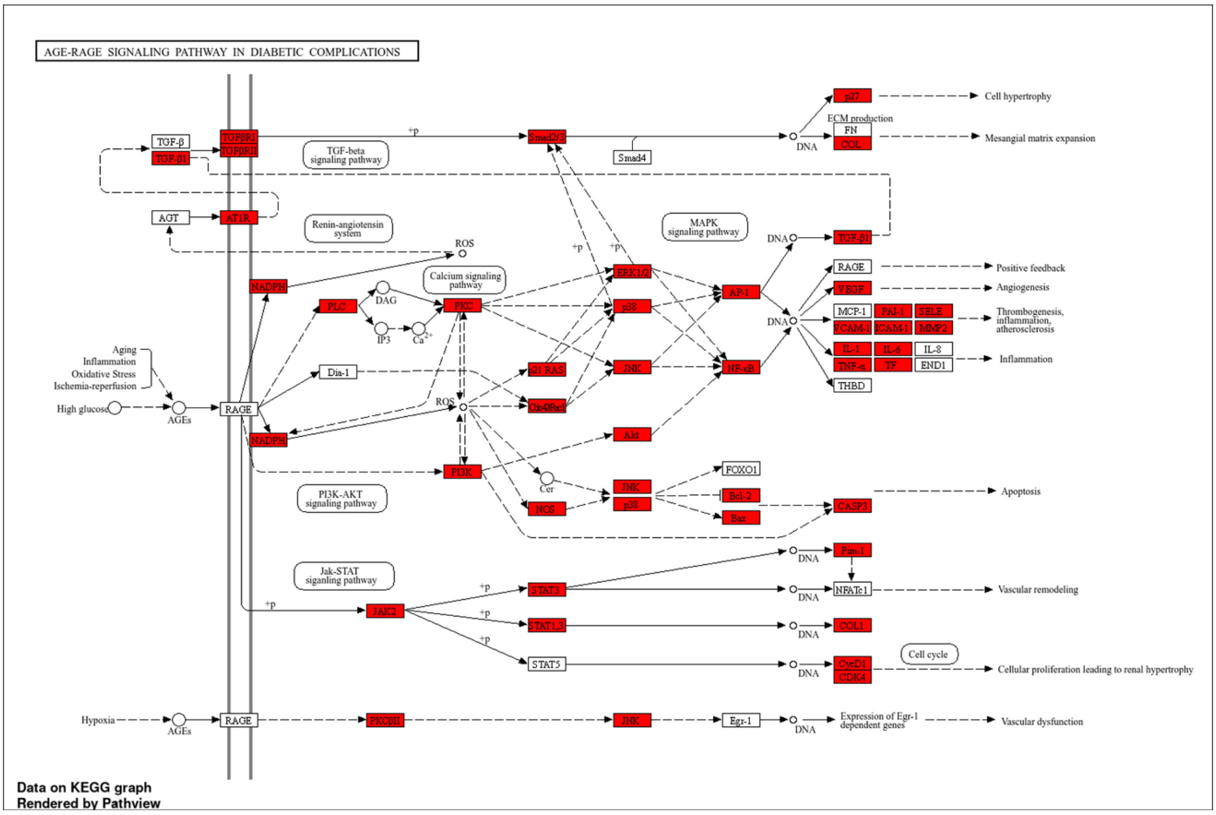

Figure S2. AGE–RAGE signaling pathway in diabetic complications identified by KEGG pathway analysis. The targets of *O. corniculata* L. compounds are marked in red.

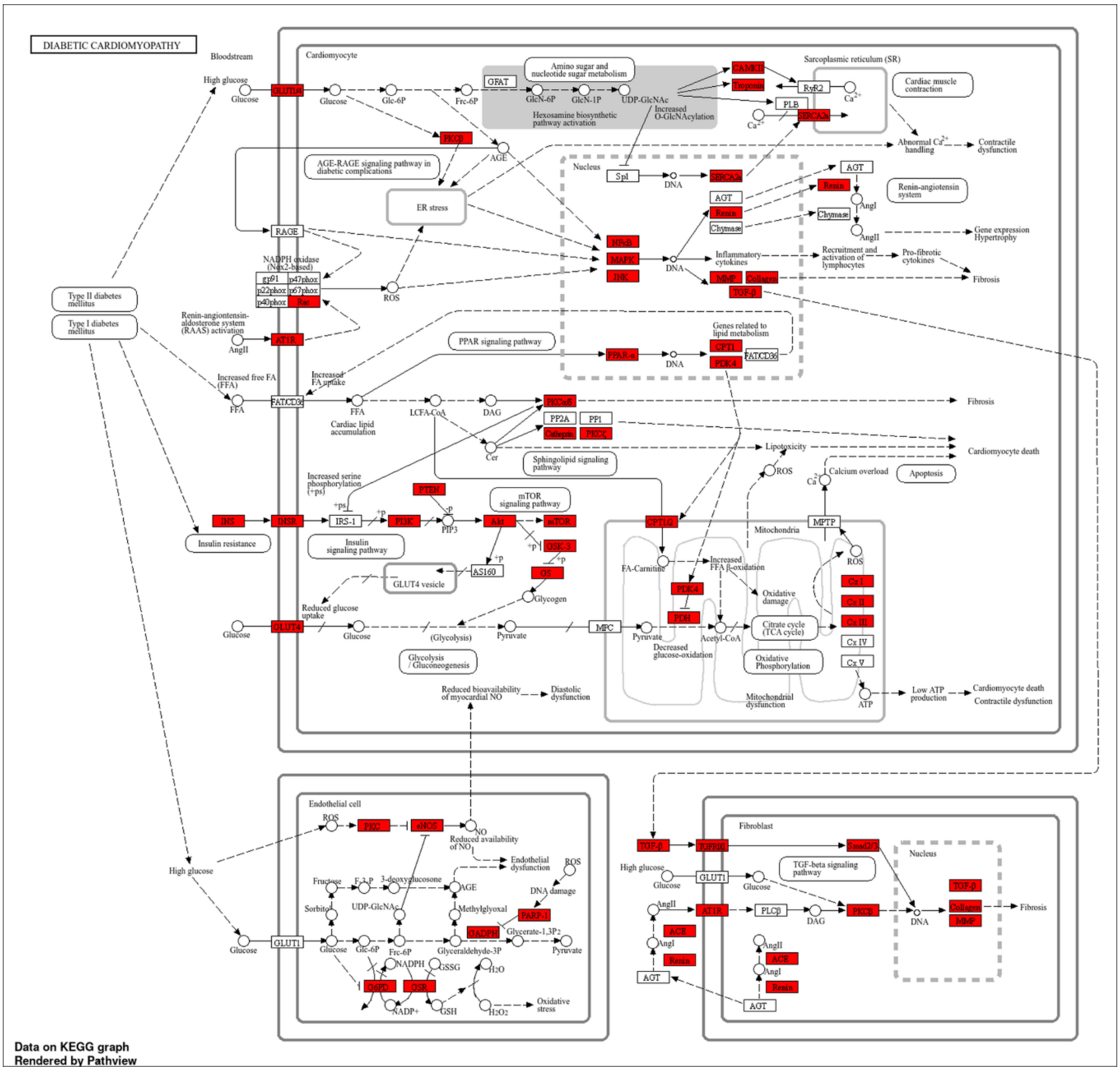

Figure S3. Cardiomyopathy pathway in diabetic complications identified by KEGG pathway analysis. The targets of *O. corniculata* L. compounds are marked in red.

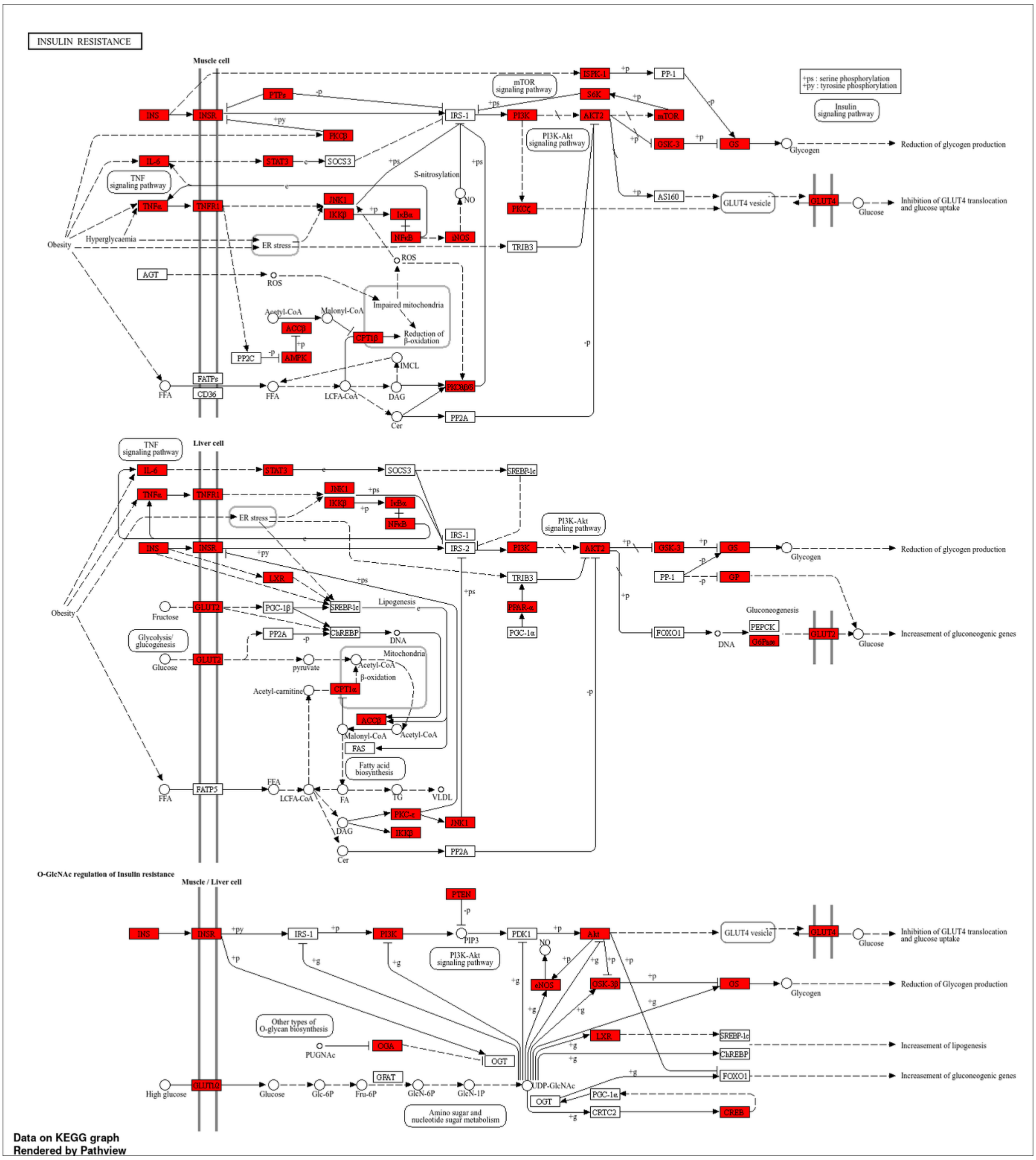

Figure S4. KEGG insulin resistance pathway. Protein targets of compounds derived from *O. corniculata* L. are highlighted in red.

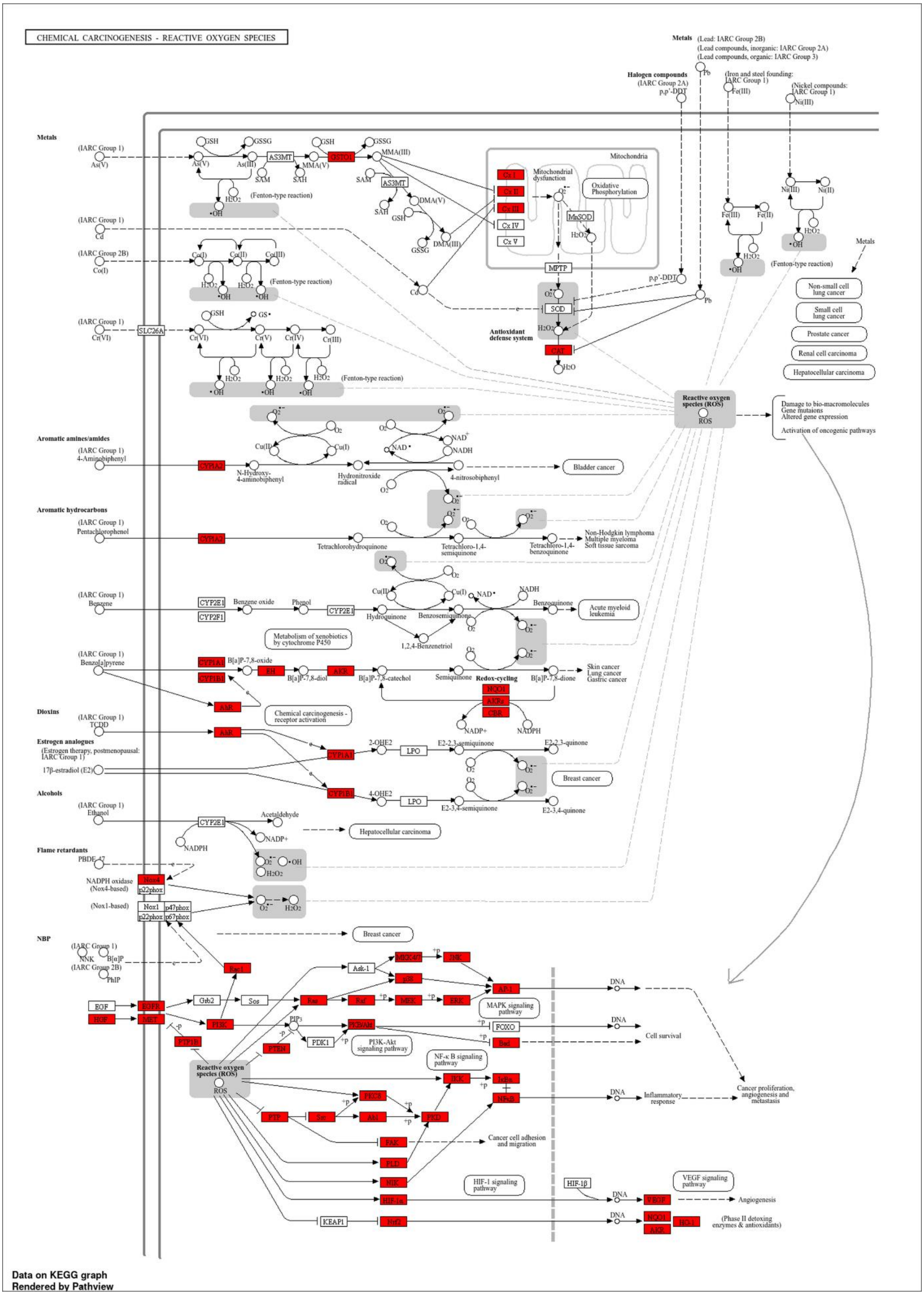

Figure S5. Chemical carcinogenesis – reactive oxygen species (ROS) pathway identified by KEGG analysis. Protein targets of compounds derived from *O. corniculata* L. are highlighted in red.
